# Mental Health Specialist Telemedicine Uptake and Patient Location

**DOI:** 10.1001/jamanetworkopen.2026.0823

**Published:** 2026-03-05

**Authors:** Jacob Jorem, Andrew D. Wilcock, Alisa B. Busch, Haiden A. Huskamp, Ateev Mehrotra

**Affiliations:** 1 Department of Health Care Policy, Harvard Medical School, Boston, Massachusetts; 2 Department of Health Services, Policy and Practice, Brown School of Public Health, Providence, Rhode Island; 3 McLean Hospital, Mass General Brigham Department of Psychiatry, Harvard Medical School, Belmont, Massachusetts

## Abstract

**Importance:**

Wide geographic disparities in mental health care use exist, particularly between rural and urban areas. Telemedicine could enable mental health specialists to reach patients who live farther away in rural communities and communities with low access to care.

**Objective:**

To examine the association of the proportion of mental health specialists’ visits delivered via telemedicine and the share of their visits to patients living in rural, low-access-to-care, or distant communities.

**Design, Setting, and Participants:**

This cohort study examined Medicare fee-for-service claims for mental health specialist services from January 1, 2018, to December 31, 2023. Specialists were categorized into quartiles based on their 2021 telemedicine uptake (lowest, 0%-40% of visits; low-middle, 41%-79%; middle-high, 80%-98%; highest, 99%-100% of visits). Data were analyzed between November 2024 and December 2025.

**Exposure:**

Telemedicine use among mental health specialists in 2021.

**Main Outcomes and Measures:**

The main outcome was the proportion of mental health specialists’ patients who lived in (1) a rural area, (2) an area with a mental health specialist shortage, (3) a different state from their specialist, and (4) a community 20 miles or more from their specialist. Differential changes in outcomes between specialists in the highest and lowest telemedicine uptake quartiles were estimated using a difference-in-differences framework. A secondary analysis examined the fraction of the changes observed due to established patients moving their residence vs new patients.

**Results:**

The cohort included 17 742 mental health specialists categorized into quartiles based on their telemedicine uptake in 2021. Compared with 2018 and specialists in the lowest telemedicine quartile, specialists with the highest telemedicine use had 0.88 percentage points (95% CI, 0.35-1.39 percentage points) more visits with rural patients in 2023. Similar small changes were observed in the fraction of visits with patients living in mental health specialist shortage areas, in a different state from their specialist, and living 20 miles or more away from their specialist. Specialists with higher telemedicine use visited differentially fewer new patients by 2023 than those with lower use (−3.55 percentage points [95% CI, −5.73 to −1.38 percentage points]).

**Conclusions and Relevance:**

This cohort study found that greater telemedicine uptake was associated with only small increases in the share of visits to patients in rural, low-access-to-care, or distant communities. Tailored policy interventions may be needed for telemedicine to reach its potential of improving mental health care of individuals with the greatest difficulty accessing it in their local community.

## Key Points

**Question:**

What is the association between the proportion of visits delivered via telemedicine by mental health specialists and the percentage of patients living in rural, low-access-to-care, or distant communities?

**Findings:**

In this cohort study of 17 742 mental health specialists serving a Medicare fee-for-service population, greater telemedicine uptake was associated with small increases in the percentage of patients living in rural, low-access-to-care, or distant communities between 2018 and 2023. A sizeable fraction of the observed changes was accounted for by established patients moving farther away (vs strictly by seeing more new patients from these communities).

**Meaning:**

These findings suggest that telemedicine uptake is not associated with substantial increases in the mental health treatment of patients in rural, low-access-to-care, or distant communities, highlighting the need for tailored policy interventions.

## Teaser

This cohort study examines the association between the proportion of telemedicine visits delivered by mental health specialists and the share of their visits among Medicare fee-for-service patients living in rural, low-access-to-care, or distant communities.

## Introduction

Wide geographic disparities in the use of mental health care exist in the US, particularly between urban and rural residents.^1,2^ These disparities are, in part, driven by the limited availability of local mental health specialists in many communities.^3^ For example, approximately 60% of US counties, including 80% of rural counties, have no practicing psychiatrists.^4,5^

Telemedicine could help to address these access barriers.^6,7^ The onset of the COVID-19 pandemic spurred dramatic growth in the use of telemedicine for mental health treatment.^8^ In 2021, more than one-third of mental health visits were conducted via telemedicine,^9^ and by 2022, 13% of mental health specialists had switched to a telemedicine-only model.^10^ Policymakers had hoped that the adoption of telemedicine would allow mental health specialists to treat geographically distant patients in rural and low-access-to-care communities, thereby expanding access to care.^11,12^ For example, Mehmet Oz, MD, MBA, Director of the Centers for Medicare & Medicaid Services, stated during a 2025 senate hearing, “Telepsychiatry, which is for mental health services, [is] vital in places like Montana [and] especially where we have many people who are suffering from undiagnosed mental health illnesses and there’s no place to go in a consistent fashion to get those therapies.”^13^

However, it is also possible that telemedicine has not led to mental health specialists treating more patients in rural communities.^14,15,16^ Given the high demand for care, mental health specialists may continue to preferentially deliver care to patients from their local community. Furthermore, if telemedicine allows established patients to stay in care longer, this might limit access for new patients who live in communities with mental health specialist shortages. Patient barriers may further limit specialists’ ability to reach those living in rural and low-income communities. Rural populations are less likely to use telemedicine partly due to inadequate broadband infrastructure.^17,18,19,20,21^ Additional patient barriers include privacy concerns, language barriers, and low levels of digital literacy.^14,17,22^

To our knowledge, no prior work has assessed the association between telemedicine uptake and the geography of a mental health specialist’s patient population. We examined a cohort of mental health specialists serving Medicare fee-for-service beneficiaries from 2018 to 2023 and grouped them by their uptake of telemedicine. Before the COVID-19 pandemic, telemedicine played a minimal role in the delivery of mental health services.^23^ The dramatic expansion beginning in March 2020 created a natural experiment in which some mental health specialists switched to a fully virtual model while others adopted telemedicine to a lesser degree. We used this variation to examine the association between the geographic reach of mental health specialists with higher vs lower levels of telemedicine adoption. Using a difference-in-differences framework,^24^ we measured differential changes in the fraction of their visits that were for patients living in rural areas, in mental health shortage areas, in another state, or 20 miles or more from their office.

## Methods

This cohort study examined 100% of Medicare fee-for-service program data from January 1, 2018, to December 31, 2023, to identify mental health specialists (psychiatrists, psychologists, clinical psychologists, neuropsychiatrists, licensed clinical social workers, and psychiatric mental health nurse practitioners^25^) actively practicing for the entire period. The study was approved by the Harvard Medical School Institutional Review Board with a waiver of informed consent because the use of deidentified administrative records presented little risk to privacy. The study followed the Strengthening the Reporting of Observational Studies in Epidemiology (STROBE) reporting guideline.

Each outpatient visit was categorized as telemedicine (video or audio only) or in-person. We restricted the cohort to specialists who had not changed practices or physical location during the study period to minimize the effect of changes in practice location. Definitions of active practice, visits, telemedicine, and specialties and how exclusions were operationalized are provided in the eMethods and eFigure 1 in Supplement 1.

### Uptake of Telemedicine

Because our goal was to assess whether telemedicine uptake led to changes in mental health specialists’ patient population, we excluded those who were already using a substantial amount of telemedicine before the pandemic (>5% of their outpatient visits in 2018 [removing <2% of visits]). We also excluded specialists with no telemedicine visits in 2020 (which included the early months of the pandemic when in-person visits were avoided by many practices for safety reasons) because we were concerned that these specialists were erroneously coding their telemedicine visits as in-person visits. We then categorized eligible specialists into the following 4 approximate quartiles based on the percentage of their outpatient mental health visits delivered via telemedicine in 2021: lowest (0%-40%), low-middle (41%-79%), middle-high (80%-98%), and highest (99%-100%) (eFigure 2 in Supplement 1). We chose 2021 to categorize specialists as it represented a more stable period than 2020, which saw outpatient treatment disruptions due to the COVID-19 pandemic. Additionally, 2021 better reflects longer-term telemedicine use following the pandemic onset (eFigure 3 in Supplement 1).

### Characteristics of Mental Health Specialists

For each mental health specialist, we captured their training (psychiatrist, psychologist, social worker, psychiatric mental health nurse practitioner), total number of outpatient mental health visits in 2021, practice size measured as the number of unique clinicians billing under the same tax identification number, practice rurality based on rural-urban commuting area code^26^ for their 5-digit zip code (metropolitan, micropolitan, small town, or rural), and the census region of their practice zip code (Northeast, Midwest, South, or West).^27^ Individual-level demographic characteristics of the specialists were not collected or analyzed.

### Visit Characteristics

We captured the following patient characteristics for each visit from the Master Beneficiary Summary File: age, sex, disability as the original reason for Medicare entitlement, low income (as captured by Medicaid dual eligibility), number of chronic conditions (of 30 total conditions captured by the Chronic Conditions Warehouse algorithms), substance use disorder (defined as *International Statistical Classification of Diseases, Tenth Revision* [*ICD-10*] codes F10-F19, excluding F17 for smoking cessation), and the presence of a serious mental illness diagnosis (defined as *ICD-10* codes F20-F29 [schizophrenia, schizotypal, delusional, and other nonmood psychotic disorders] and *ICD-10* codes F30 and F31.0-F31.7 [bipolar I disorder]). Data on race and ethnicity were not collected or analyzed as these data are not consistently collected by Medicare. The patient’s geographic location for a given visit was based on their mailing address zip code on the claim. Details on how these variables were captured, including diagnosis codes, are provided in the eMethods in Supplement 1.

### Outcomes

We captured 4 measures of geographic reach. Each outcome was captured at the visit level and then aggregated across all visits in a given period for each group of specialists. This approach allowed us to measure, for example, the percentage of visits by specialists with the highest telemedicine uptake in 2023 to rural patients.

The first outcome captured whether the patient’s zip code was located in a mental health specialist shortage area, defined as a county in 2019 without a psychiatrist’s practice zip code in our data. All psychiatrists treating Medicare fee-for-service patients, including those not meeting our inclusion criteria, were considered for this measure. We used the availability of psychiatrists instead of all mental health specialists, given prior literature examining geographic variation in the availability of psychiatrists (more details provided in the eMethods in Supplement 1).^5^ The second outcome was whether the patient’s zip code was located in a rural area, defined as a nonmetropolitan zip code (rural-urban commuting area codes 4-10).^27^ The third outcome indicated whether the patient’s zip code was in a different state from the specialist’s zip code of practice. The fourth outcome was whether the distance between the patient’s residence and the specialist’s practice location was 20 miles or more. We calculated this distance using the longitude and latitude coordinates of the centroids of the patient and specialist’s 5-digit zip codes.

To better understand the context of our outcomes, we also examined the share of a specialist’s patients who were new (vs established). A new patient was defined as one who the specialist had not seen in the previous 2 years. This outcome was analyzed at the specialist-patient-year level.

### Statistical Analysis

The data analyses were performed between November 2024 and January 2025. Univariate analyses were used to describe visit characteristics by specialist telemedicine use. We calculated monthly trends in each outcome for the 4 telemedicine use groups and plotted unadjusted trends from 2018 to 2023. To show the change in each outcome over time, we also plotted each monthly time series point compared with its value in January 2018, the first month of our study period. For example, if the mean percentage of visits for rural patients in the lowest telemedicine group was 10% in January 2018 and 15% by June 2023, the change for this group in June 2023 would be 5 percentage points (ie, 15% minus 10%).

Next, we used a difference-in-differences framework, using a linear regression model to estimate adjusted differential changes in our outcomes by year and telemedicine group. The unit of the analysis was the visit, and the outcomes were binary (yes or no). We included a fixed effect for each specialist to capture any unobserved, inherent differences between specialists that may affect our outcomes, a fixed effect for each year to capture the secular trend in our outcomes, and interactions between the year and the telemedicine group to capture the differential change each year by the telemedicine group. The SEs were clustered at the practice level (practices were identified by tax identification number). We used a linear model because, especially for large sample sizes, the estimates produced by such models are typically consistent and unbiased, and the coefficients in this context are easier to interpret. For example, a coefficient of 0.0012 would be interpreted as a change of 0.12 percentage points in the percentage of visits with a given outcome across a set of specialists.

By including our telemedicine effects by year, our model allowed us to measure the evolution of the association between increased telemedicine use and changes in our outcomes. Between 2018 and 2019, we expected no significant differences by telemedicine group (ie, no preperiod trend) as telemedicine was not commonly used until early 2020. Thereafter, in the years 2020, 2021, 2022, and 2023, we captured any differential changes by telemedicine group in each year and whether those changes grew over time. To accommodate the multiple comparisons we made in each model (15 comparisons), we used Bonferroni-corrected 95% CIs to evaluate the significance on our telemedicine group-by-year interactions.

Since all specialists in our cohort, by definition, worked from 1 practice location during the study period, the geographic reach of their visits would primarily be driven by existing patients moving or by seeing new patients living closer or farther away. To examine which of these scenarios could better explain the observed changes, we reran our analyses for which each patient’s zip code was replaced with their first zip code with a specialist. This approach removed variation due to changes in patient address. After estimating our model while controlling for patients who moved, we contrasted these coefficients with our main model findings to better understand the role of patient movement in influencing the observed changes.

We also conducted several sensitivity analyses. First, we relaxed exclusion criteria, allowing specialists who moved locations or practices to remain in the cohort. Second, we used alternative cutoffs for telemedicine delivery quartiles (0%-15% minimal use, 16%-50% half or less use, 51%-89% majority use, and 90%-100% high use). Third, we tested other distance thresholds (10 and 30 miles) and conducted a secondary analysis in which we did not use a threshold of 20 miles and instead examined the distribution of distances across all visits using kernel density distributions. All statistical analyses were performed using Stata, version 18.0 (StataCorp LLC).

## Results

The cohort included 17 742 mental health specialists with 25.0% of the sample categorized into each of the 4 quartiles based on telemedicine use in 2021 (Table): 4437 in the lowest (0%-40%), 4434 in the low-middle (41%-79%), 4441 in the middle-high (80%-98%), and 4430 in the highest (99%-100%) groups. Social workers made up the largest percentage of specialists (44.5%), followed by psychologists (35.0%), psychiatrists (14.7%), and psychiatric nurse practitioners (5.8%).

**Table.** Characteristics of Mental Health Specialists and Their Patient Panels in 2021 by Telemedicine Use

Characteristic	Lowest quartile (0%-40%)	Low-middle quartile (41%-79%)	Middle-high quartile (80%-98%)	Highest quartile (99%-100%)	Absolute SMD (lowest vs highest)^a^
**Specialist characteristics, No. (%)**					
No. of specialists	4437	4434	4441	4430	NA
Mental health visits per specialist, mean (SD)	279.0 (284.5)	273.0 (259.8)	286.8 (291.9)	246.6 (226.1)	0.126
Telemedicine share, mean (SD)	16.4 (0.1)	61.4 (0.1)	91.6 (0.1)	99.9 (0)	8.685
Type of mental health specialist					
Psychiatrist	621 (14.0)	672 (15.2)	764 (17.2)	559 (12.6)	0.041
Psychologist	1531 (34.5)	1422 (32.1)	1518 (34.2)	1742 (39.3)	0.100
Social worker	1984 (44.7)	2051 (46.3)	1877 (42.3)	1976 (44.6)	0.002
Psychiatric nurse practitioner	301 (6.8)	289 (6.5)	282 (6.4)	153 (3.5)	0.152
Practice size, No. of clinicians					
1	2690 (60.6)	2511 (56.6)	2588 (58.3)	3200 (72.2)	0.248
2-9	873 (19.7)	791 (17.8)	720 (16.2)	534 (12.1)	0.210
10-20	361 (8.1)	491 (11.1)	473 (10.7)	354 (8.0)	0.005
21-49	198 (4.5)	269 (6.1)	289 (6.5)	163 (3.7)	0.040
≥50	319 (7.2)	381 (8.6)	375 (8.4)	179 (4.0)	0.137
Rurality					
Metropolitan	3815 (86.0)	3916 (88.3)	4106 (92.5)	4213 (95.1)	0.315
Micropolitan	417 (9.4)	358 (8.1)	240 (5.4)	137 (3.1)	0.263
Small town	154 (3.5)	120 (2.7)	71 (1.6)	56 (1.3)	0.146
Rural	51 (1.2)	40 (0.9)	24 (0.5)	24 (0.5)	0.066
Census region					
Northeast	1129 (25.5)	1491 (33.6)	1811 (40.8)	2243 (50.6)	0.537
Midwest	1138 (25.7)	1154 (26.0)	878 (19.8)	645 (14.6)	0.279
South	1402 (31.6)	1109 (25.0)	1074 (24.2)	792 (17.9)	0.322
West	768 (17.3)	680 (15.3)	678 (15.3)	750 (16.9)	0.010
**Patient panel characteristics, mean (SD)^b^**					
Age, y	64.9 (8.4)	64.1 (8.2)	64.9 (8.3)	66.7 (7.8)	0.230
Sex					
Female	68.0 (21.1)	69.5 (21.3)	71.2 (21.9)	71.8 (24.4)	0.166
Male	32.1 (21.1)	30.5 (21.3)	28.8 (21.9)	28.3 (24.4)	
Low income^c^	28.8 (28.9)	30.9 (29.6)	28.6 (29.8)	22.5 (28.2)	0.223
Disability^d^	47.5 (28.4)	49.6 (29.0)	47.0 (29.8)	40.5 (30.1)	0.239
No. of chronic conditions^e^	454.7 (1.4)	442.5 (1.3)	447.2 (1.3)	444.6 (1.4)	0.072
Substance use disorder diagnosis	2.9 (9.8)	3.1 (8.9)	2.8 (8.5)	1.7 (6.6)	0.151
Serious mental illness diagnosis^f^	13.9 (19.5)	14.9 (19.0)	14.3 (18.8)	10.7 (17.6)	0.173

Abbreviations: *ICD-10*, *International Statistical Classification of Diseases, Tenth Revision*; NA, not applicable; SMD, standardized mean difference.

^a^ Values larger than 0.1 indicate a sizeable difference.

^b^ First, the average patient characteristics of visits per specialist were summarized, and then the mean was calculated across the specialists in each telemedicine group.

^c^ Identified through dual eligibility with Medicaid at the time of their visit.

^d^ The original reason for Medicare entitlement was disability.

^e^ Included the number of chronic conditions (of a total of 30 conditions captured by the Chronic Conditions Warehouse algorithms), including depression, bipolar disorder, or other depressive mood disorders.

^f^ Defined as schizophrenia, schizotypal, delusional, and other nonmood psychotic disorders (*ICD-10* codes F20-F29) or bipolar I disorder (*ICD-10* codes F30, F31.0-F31.7) in any diagnosis position on the associated carrier claim for the visit.

Specialist characteristics varied across telemedicine groups. For example, compared with those in the lowest telemedicine group, specialists in the highest group were more likely to be solo practitioners (72.2% vs 60.3%) and based in urban areas (95.1% vs 86.0%). Patient panels also differed, in which the highest telemedicine group compared with the lowest group treated fewer patients with low incomes (mean [SD], 22.5% [0.3%] vs 28.8% [0.3%]), disabilities (mean [SD], 40.5% [0.3%] vs 47.5% [0.3%]), and serious mental illnesses (mean [SD], 10.7% [0.2%] vs 13.9% [0.2%]).

**Figure 1.**
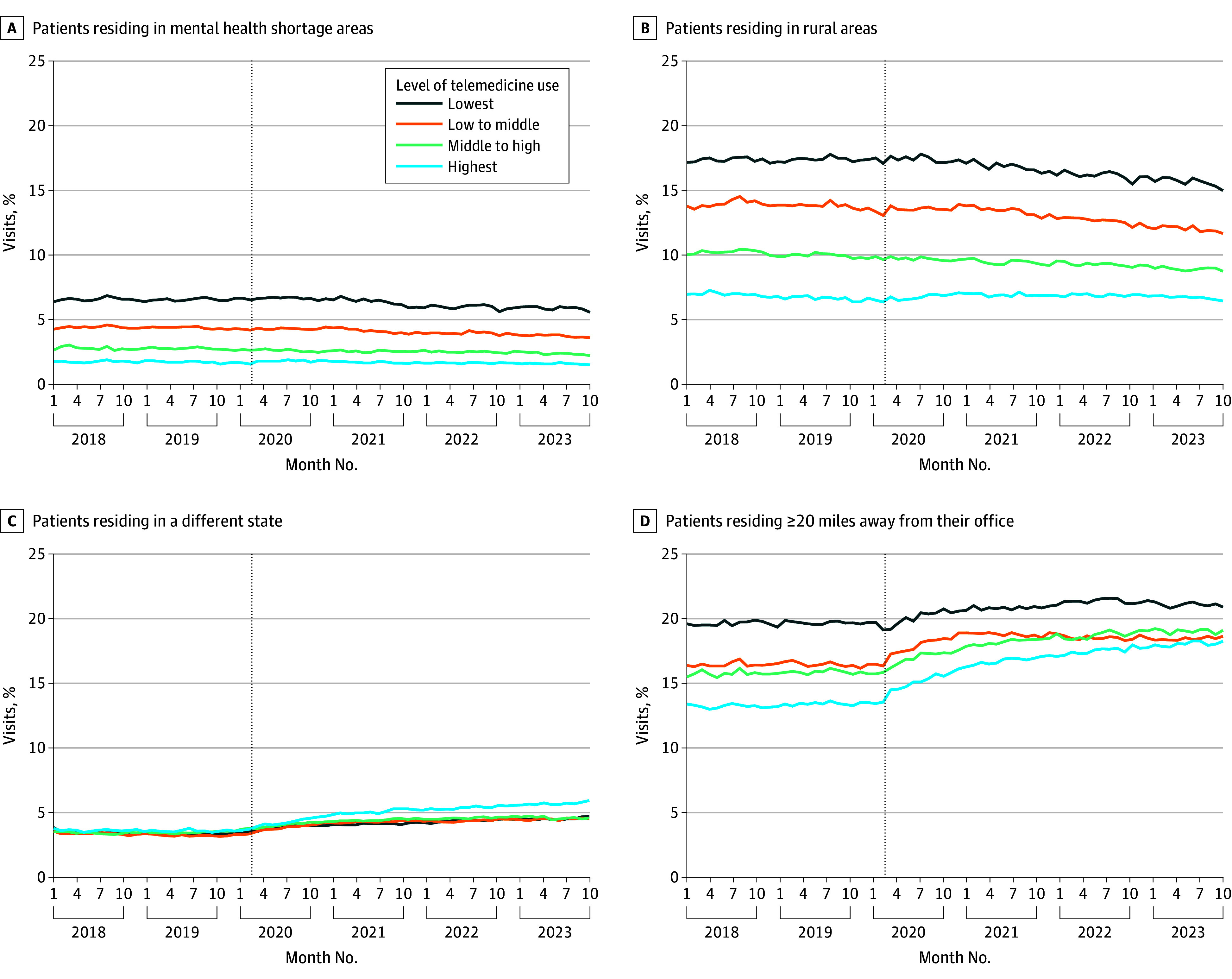
Line Graphs of Monthly Unadjusted Trends in Outcomes From 2018 to 2023 by Mental Health Specialists’ Level of Telemedicine Use in 2021 The dotted vertical lines indicate the start of the COVID-19 pandemic in March 2020.

In the prepandemic period (2018-2019), there were notable baseline differences across the outcomes (Figure 1). For example, specialists in the highest telemedicine group compared with the lowest group had a lower percentage of visits with patients living in mental health shortage areas (2% vs 6%), in rural areas (7% vs 17%), and 20 miles or more away (13% vs 20%). Levels were stable before the pandemic.

### Trends in Outcomes

Following the onset of the pandemic in March 2020, greater telemedicine adoption was associated with modest differences in our 4 geographic reach outcomes (Figure 1). For example, visits with patients living 20 miles or more away increased 5.1 percentage points from 13.1% in December 2018 to 18.2% in December 2023 in the highest telemedicine group while it increased 1.3 percentage points from 19.6% and 20.9%, respectively, in the lowest telemedicine group (Figure 1D). Moreover, all groups of specialists showed post-COVID increases in the percentage of visits for patients residing 20 miles or more away and in different states (Figure 1C). To better illustrate their size, these trends are presented as changes compared with January 2018 in eFigure 4 in Supplement 1.

Changes in distance between specialists and patients were also found in our examination of kernel densities, in which we examined the distribution of distances in 2018 and 2023. Among specialists in the lowest telemedicine group, there was no discernible difference. In contrast, for specialists with the highest telemedicine adoption, we found a slight increase by 2023 (eFigure 5 in Supplement 1).

### Differential Changes in Outcome by Telemedicine Group

**Figure 2.**
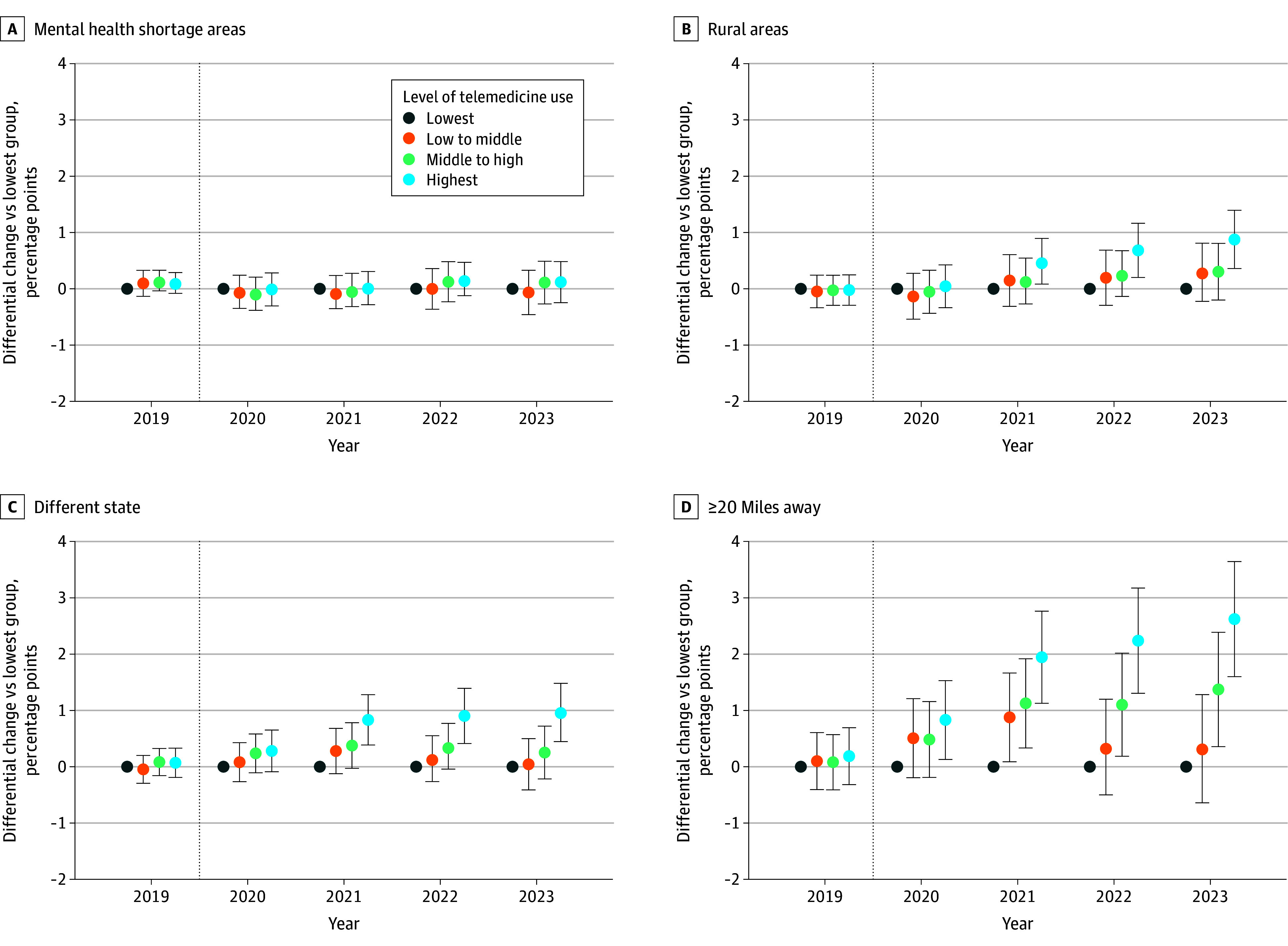
Dot Plots of Differential Changes in the Percentage of Visits With Patients in a Given Geographic Area Over Time by Mental Health Specialists’ Level of Telemedicine Use Plots show the differential changes in the preperiod between 2018 and 2019, in which there was little to no telemedicine use and no significant or sizeable association of future telemedicine use (defined in 2021) with the outcomes (ie, there was no evidence of preperiod differential trends by telemedicine group for any of the outcomes). D, The percentage of visits with patients residing 20 or more miles away differentially increased among the higher telemedicine groups (vs the lowest group) each year following the start of the COVID-19 pandemic in March 2020 and increased more with greater telemedicine use. By 2023, visits at least 20 miles away among the highest telemedicine group had increased 2.62 percentage points (95% CI, 1.60-3.64 percentage points) more than in the lowest telemedicine group. Marginal effect estimates in each year show the difference in changes (vs 2018) vs the lowest group (represented as 0 in all years). Full model estimates (absolute and relative) are included in eTables 1 to 4 in Supplement 1. Error bars indicate the Bonferroni-corrected 95% CIs, and the dotted vertical lines separate the prepandemic year from subsequent pandemic years.

These unadjusted differences were reflected in our difference-in-differences models (Figure 2; eTables 1-4 in Supplement 1). Here, we focus on the differences between 2018 and 2023 for mental health specialists in the lowest vs highest telemedicine groups. Compared with specialists in the lowest telemedicine group, specialists in the highest group showed increases of 0.12 percentage points (95% CI, −0.24 to 0.48 percentage points) in visits with patients living in shortage areas; for patients living in rural areas, another state, or 20 or more miles away, increases were 0.88 percentage points (95% CI, 0.35-1.40 percentage points), 0.95 percentage points (95% CI, 0.42-1.48 percentage points), and 2.62 percentage points (95% CI, 1.60-3.64 percentage points), respectively. The findings were similar when we used alternative cutoffs to define our telemedicine groups, relaxed some of our exclusion criteria, included a larger cohort of mental health specialists, limited our analytic data to 1 observation per patient-specialist per year, or used different distance cutoffs (eFigures 6-9 in Supplement 1).

### Change in Estimates From Patients Moving

**Figure 3.**
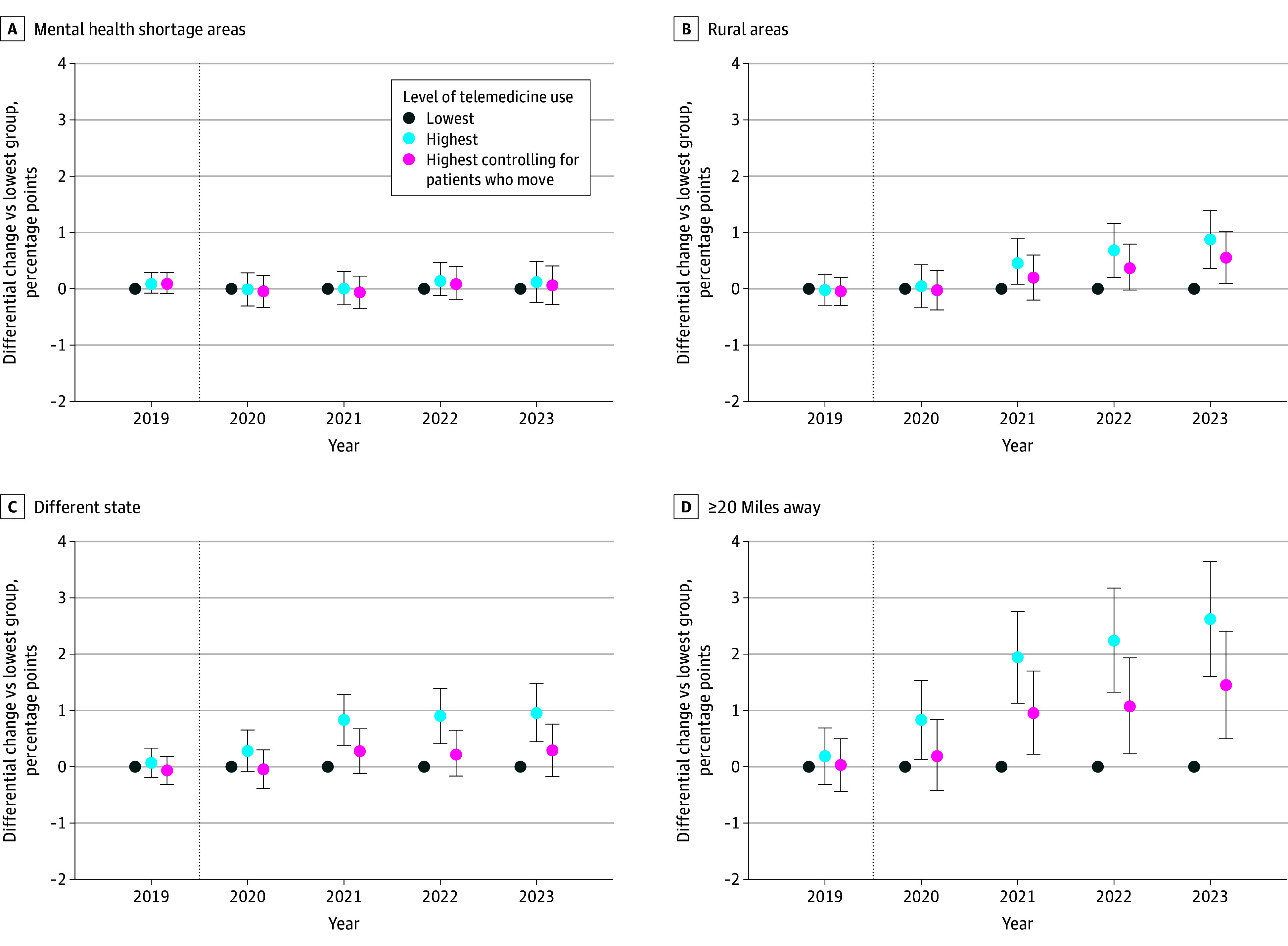
Dot Plots of Differential Changes in the Percentage of Visits With Patients in a Given Geographic Area Over Time Between Mental Health Specialists With the Highest and Lowest Telemedicine Use, With and Without Controlling for Patients Who Moved To simplify the comparison, only coefficients from the year-telemedicine group interactions are shown for the highest group from each model. Full model coefficients are included in eTables 1 to 4 in Supplement 1. Error bars indicate the 95% CIs, and the dotted vertical lines separate the prepandemic year from subsequent pandemic years.

To control for patients moving during our study period, we fixed the patient’s address from their first visit with a mental health specialist and then remeasured changes in our outcomes. Patients moving explained the majority of the differential increase in visits from patients in different states (0.29 percentage points [95% CI, −0.02 to 0.60 percentage points] after controlling for moves) and nearly one-half of the differential increase in visits for patients residing 20 miles or more away (1.45 percentage points [95% CI, 0.50-2.40 percentage points] after controlling for moves) (Figure 3; eTables 1-4 in Supplement 1).

### Percentage of New vs Established Patients

**Figure 4.**
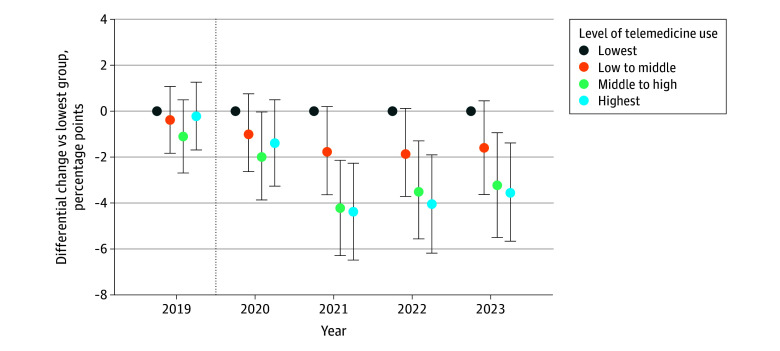
Dot Plots of Differential Changes in the Percentage of New Patients Over Time by Mental Health Specialists’ Level of Telemedicine Use Data were analyzed at the patient-specialist-year level, in which each patient-specialist combination was only counted once per year. Error bars indicate the 95% CIs, and the dotted vertical lines separate the prepandemic year from subsequent pandemic years.

Patients were identified as new if the specialist did not have a bill (a carrier claim) with the patient in the previous 2 years; for all subsequent visits within 2 years, the patient was considered established. We found that specialists with higher telemedicine use had fewer new Medicare fee-for-service patients (−3.55 percentage points [95% CI, −5.73 to −1.38 percentage points]) compared with specialists with lower telemedicine use before and after the onset of the pandemic (Figure 4).

## Discussion

This cohort study found that greater telemedicine adoption among mental health specialists was associated with only small differences in the percentage of patients who lived in rural, low-access-to-care, or distant communities. Furthermore, the observed small differences were accounted for by established Medicare fee-for-service patients moving farther away from their specialists rather than strictly by specialists seeing more new patients from those communities. Together, our findings suggest that despite the dramatic shift from in-person care to telemedicine among mental health specialists, telemedicine uptake was not associated with a substantive change in the geographic distribution of patient panels of mental health specialists in the Medicare population.

Several factors may help explain these findings between telemedicine adoption and treating patients in rural, low-access-to-care, or distant communities. Specialists with high telemedicine adoption were predominantly based in urban areas and already served fewer Medicare fee-for-service patients with lower incomes and living in rural areas before the COVID-19 pandemic onset. Without changes to underlying referral networks, often through word of mouth and embedded within local communities, rural patients may have had difficulty identifying specialists outside of their communities who were willing to treat via telemedicine. Given that mental health care use has increased since after the pandemic^19^ and the high demand for mental health specialists, many specialists may have had little capacity to treat new patients in rural communities. As we noted, limited broadband and low digital literacy may limit specialists’ ability to reach patients in rural communities. Awareness of available telemedicine services in rural communities may also be limited.^28^ In addition, state coverage and licensure regulations may restrict specialists’ ability to treat patients in other states, further limiting the potential for telemedicine to broaden its geographic reach.^29^

Paradoxically, greater telemedicine adoption may reduce specialists’ availability for new patients. Telemedicine adoption seems to benefit established patients by allowing them to remain connected with their specialists, even after moving farther away, potentially extending the duration of existing care. These findings echo previous work suggesting that telemedicine may contribute to greater continuity of care^30^ and higher treatment retention.^31^ While greater treatment retention could have positive clinical benefits if the need for specialist care persists, it may have the unintended consequence of less specialist capacity for new patients. Further work is needed on whether some patients who receive mental health specialty care could be safely transitioned to lower-intensity services, thereby freeing specialist capacity for new patients.^32,33^

Our findings suggest that greater telemedicine use alone may not substantially improve access for low-resourced communities. For telemedicine to fulfill its promise of improving access to mental health care in rural communities and those with low acces to care, tailored policy interventions may be needed. How can health policy motivate specialists to prioritize populations with low access to care located farther away? Dedicated referral pathways may link health care practitioners who work in these communities to specialists with high telemedicine adoption outside the local area. Reforms to licensure regulation may facilitate cross-state practice.^34,35^ Financial incentives may also help. Currently, reimbursement in Medicare is based on a specialist’s practice location. Reimbursement based on patient location with higher reimbursement rates for telemedicine visits with rural patients compared with urban patients may also spur change.

### Limitations

Our study had several limitations. The population was limited to Medicare fee-for-service beneficiaries, who represent only a portion of patients receiving outpatient mental health services nationally. Telemedicine use is more common among adults with greater health care needs, such as those with chronic conditions and older adults. Thus, our findings may not be generalizable to patients with commercial insurance or to specialists who either do not accept Medicare or treat very few Medicare fee-for-service patients each year. Because of our large sample size, even small changes were sometimes statistically significant. We therefore focused on what we viewed as substantive changes, although we acknowledge that this is a qualitative judgment and that others might disagree with our interpretation of what is considered substantive. Our measure of a health shortage area only reflected psychiatrists who served Medicare patients during the study period and did not capture other mental health specialists. Our dataset did not include services provided at federally qualified health centers. We also limited our analysis to specialists who did not change their practice site or location. However, when we relaxed this criterion in a sensitivity analysis, the findings were similar. Finally, our measures of geographic reach were based on patients’ home addresses. There may have been inaccuracies in the data used to capture patient locations, and they would not capture, for example, whether a patient was on vacation in another state at the time of the visit.

## Conclusions

This cohort study found little evidence that greater telemedicine uptake among mental health specialists improved access to care for patients in rural communities or communities with specialist shortages. Instead, greater telemedicine uptake was associated with only small increases in the fraction of visits for Medicare fee-for-service patients in these communities. Tailored policy interventions may be needed for telemedicine to reach its potential of improving mental health care of individuals with the greatest difficulty accessing it in their local community.

## Notes

Supplement 1. **eMethods. **More Details on Study Methods **eTable 1. **Linear Regressions of Patients Residing in Mental Health Shortage Area, Without and With Controlling for Patients Who Moved **eTable 2. **Linear Regressions of Patients Residing in Rural Areas, Without and With Controlling for Patients Who Moved **eTable 3. **Linear Regressions of Patients Residing in Different States, Without and With Controlling for Patients Who Moved **eTable 4. **Linear Regressions of Patients Residing 20 Miles or More Away, Without and With Controlling for Patients Who Moved **eFigure 1. **Flowchart of Mental Health Specialist Cohort Exclusions, 2018-2023 **eFigure 2. **Distribution of Telemedicine Use by Mental Health Specialists in 2021 **eFigure 3. **Telemedicine Share of Monthly Visits by 2021 Telemedicine Quartile Group Assignment, 2018-2023 **eFigure 4. **Monthly Unadjusted Trends in Outcomes From 2018 to 2023 by Mental Health Specialists’ Level of Telemedicine use in 2021, Shown as Percentage Point Differences Relative to January 2018 Levels **eFigure 5. **Kernel Density Plots of the Distribution of Log-Transformed Miles Between Patients and Specialists Among the Lowest and Highest Telemedicine Users in 2018 and 2023 **eFigure 6. **Differential Changes in Outcomes Between Highest and Lowest Telemedicine Groups Using Quartiles or Predefined Cutoffs **eFigure 7. **Differential Changes in Outcomes Between the Highest and Lowest Telemedicine Use Specialists, Using a Larger Sample of Specialists **eFigure 8. **Differential Changes in Outcomes Between the Highest and Lowest Telemedicine Use Specialists, Using an Analytic Sample of 1 Observation Per Patient-Specialist Per Year **eFigure 9. **Differential Changes in Patients Living 10, 20, or 30 Miles or More From Their Specialist Between Highest and Lowest Telemedicine Groups
eReferences.

Supplement 2.
Data Sharing Statement
