# Supplementary material for: Mental Health Specialist Telemedicine Uptake and Patient Location
Source: JAMA Netw Open. 2026 Mar 5;9(3):e260823. doi: 10.1001/jamanetworkopen.2026.0823 (PMC12964166; doi:10.1001/jamanetworkopen.2026.0823)
Supplement: Supplement 1. — eMethods. More Details on Study Methods eTable 1. Linear Regressions of Patients Residing in Mental Health Shortage Area, Without and With Controlling for Patients Who Moved eTable 2. Linear Regressions of Patients Residing in Rural Areas, Without and With Controlling for Patients Who Moved eTable 3. Linear Regressions of Patients Residing in Different States, Without and With Controlling for Patients Who Moved eTable 4. Linear Regressions of Patients Residing 20 Miles or More Away, Without and With Controlling for Patients Who Moved eFigure 1. Flowchart of Mental Health Specialist Cohort Exclusions, 2018-2023 eFigure 2. Distribution of Telemedicine Use by Mental Health Specialists in 2021 eFigure 3. Telemedicine Share of Monthly Visits by 2021 Telemedicine Quartile Group Assignment, 2018-2023 eFigure 4. Monthly Unadjusted Trends in Outcomes From 2018 to 2023 by Mental Health Specialists’ Level of Telemedicine use in 2021, Shown as Percentage Point Differences Relative to January 2018 Levels eFigure 5. Kernel Density Plots of the Distribution of Log-Transformed Miles Between Patients and Specialists Among the Lowest and Highest Telemedicine Users in 2018 and 2023 eFigure 6. Differential Changes in Outcomes Between Highest and Lowest Telemedicine Groups Using Quartiles or Predefined Cutoffs eFigure 7. Differential Changes in Outcomes Between the Highest and Lowest Telemedicine Use Specialists, Using a Larger Sample of Specialists eFigure 8. Differential Changes in Outcomes Between the Highest and Lowest Telemedicine Use Specialists, Using an Analytic Sample of 1 Observation Per Patient-Specialist Per Year eFigure 9. Differential Changes in Patients Living 10, 20, or 30 Miles or More From Their Specialist Between Highest and Lowest Telemedicine Groups eReferences. [file jamanetwopen-e260823-s001.pdf]

Supplemental Online Content

Jorem J, Wilcock AD, Busch AB, Huskamp HA, Mehrotra A. Mental health specialist telemedicine uptake and patient location. *JAMA Netw Open*. 2026;9(3):e260823. doi:10.1001/jamanetworkopen.2026.0823

eMethods. More Details on Study Methods

eTable 1. Linear Regressions of Patients Residing in Mental Health Shortage Area, Without and With Controlling for Patients Who Moved

eTable 2. Linear Regressions of Patients Residing in Rural Areas, Without and With Controlling for Patients Who Moved

eTable 3. Linear Regressions of Patients Residing in Different States, Without and With Controlling for Patients Who Moved

eTable 4. Linear Regressions of Patients Residing 20 Miles or More Away, Without and With Controlling for Patients Who Moved

eFigure 1. Flowchart of Mental Health Specialist Cohort Exclusions, 2018-2023

eFigure 2. Distribution of Telemedicine Use by Mental Health Specialists in 2021

eFigure 3. Telemedicine Share of Monthly Visits by 2021 Telemedicine Quartile Group Assignment, 2018-2023

eFigure 4. Monthly Unadjusted Trends in Outcomes From 2018 to 2023 by Mental Health Specialists' Level of Telemedicine use in 2021, Shown as Percentage Point Differences Relative to January 2018 Levels

eFigure 5. Kernel Density Plots of the Distribution of Log-Transformed Miles Between Patients and Specialists Among the Lowest and Highest Telemedicine Users in 2018 and 2023

eFigure 6. Differential Changes in Outcomes Between Highest and Lowest Telemedicine Groups Using Quartiles or Predefined Cutoffs

eFigure 7. Differential Changes in Outcomes Between the Highest and Lowest Telemedicine Use Specialists, Using a Larger Sample of Specialists

eFigure 8. Differential Changes in Outcomes Between the Highest and Lowest Telemedicine Use Specialists, Using an Analytic Sample of 1 Observation Per Patient-Specialist Per Year

eFigure 9. Differential Changes in Patients Living 10, 20, or 30 Miles or More From Their Specialist Between Highest and Lowest Telemedicine Groups

eReferences

This supplemental material has been provided by the authors to give readers additional information about their work.

© 2026 Jorem J et al. *JAMA Network Open*.

© 2026 Jorem J et al. *JAMA Network Open*.

eMethods. More Details on Study Methods

The following provides more details about the methods we used to create our study sample and variables.

Creation of Cohort of Mental Health Specialists

We identified mental health specialists in the 2018–2023 100% Medicare Part B Carrier line files using the Centers for Medicare & Medicaid Services (CMS) specialty codes for psychiatrists, psychologists, clinical psychologists, neuropsychiatrists, and licensed clinical social workers (codes 26, 62, 68, 86, and 80, respectively), as well as through National Provider Identifiers (NPIs) for psychiatric mental health nurse practitioners following prior work.[1] To restrict the cohort to those who were in continuous practice and had not changed practices or physical location in this period, we first examined all Carrier line services delivered by these specialists with an outpatient mental health Current Procedural Terminology (CPT) code.

The following table lists the specific CPT/ Healthcare Common Procedure Coding System (HCPCS)[1] codes we included as "mental health services":

MH/SUD Services Category	CPT/HCPCS Codes
Crisis intervention services	90839, H0007, H2011, S9484, S9485
Assessments, E&M services, substance use disorders medication services	90791, 90792, 90801, 90802, 90863, 99058, 99201-99205, 99211-99215, 99241-99245, 99341-99345, 99347, 99348, 99349, 99350, 99382, 99383, 99384, 99392, 99393, 99394, 99483,99495, 99496, G0155, G0438, G0439, G0463, G0466, G0467, G0469, G0470, G0505, H0001, H0002, H0014, H0016, H0020, H0022, H0023, H0046, H1011, H2000, H2010, T1007, T1011, T1015, T0123, T1040, G0402
Psychotherapy services	Individual or Family: 90804-90815, 90820, 90832-90838, 90845, 90846, 90847-90849, 90865, 90875, 90876, 90880, 99510, H0004, H2019, Group: 90853, 90857
Supportive psychosocial services	S9127, S9482, H2028, H2029, T1024

[1] Codes were based on work done in Busch AB, Huskamp HA, Raja P, Rose S, Mehrotra A. Disruptions in care for Medicare beneficiaries with severe mental illness during the COVID-19 pandemic. JAMA Netw Open. 2022;5(1):e2145677. doi:10.1001/jamanetworkopen.2021.45677

© 2026 Jorem J et al. *JAMA Network Open*.

Screening and preventative counseling or services	99395-99397, 99408, 99409, 99411, 99483, G0396, G0397, G0402, G0438, G0439, G0442, G0443, H0049
Codes specific for telemedicine visits	Audio only: 98966-98968<br>Video: G2025 or outpatient visit with modifier code (modifiers GQ, GT or 95)

Multiple services delivered by a specialist to the same patient on the same day were considered one mental health visit. Then, mental health visits were only considered outpatient if the place of service codes for the visit were limited to telemedicine, office, home, assisted-living, or group home (codes 02, 10, 11, 12, 13, 14, respectively).

Specialists were included if they provided at least 30 outpatient mental health visits each year between 2018 and 2023 (what we referred to as “active practice”). We removed specialists with more than one practice (identified using their Tax Identification Number (TIN)) or more than one physical location (identified using the specialist’s 5-digit zip code) over this same period. We also excluded specialists who did not bill any telemedicine visits in 2020, a period when in-person services were largely infeasible. Given the complexity of telemedicine billing codes, we suspect that some specialists may have provided telemedicine without using the special billing codes. To minimize this source of measurement error, we limited our analysis to specialists who billed at least once for telemedicine in 2020. See **eFigure 1** below for a cohort sample diagram demonstrating the impact our exclusions had on the size of our study sample.

Defining the Mental Health Specialist Shortage Area Measure

In creating the mental health specialist shortage area measure, we echoed HRSA’s measure of mental health professional shortage areas (HPSAs), which uses the supply of psychiatrists in a community as a way to categorize geographic areas as HPSA vs. not. The major criticism of HPSAs is that they include all psychiatrists, even though roughly half of psychiatrists do not accept Medicare, and they include many who are retired. To address this criticism, we assessed whether there was a psychiatrist in the county who actually provided care to a fee-for-service Medicare beneficiary. Another version of this would be to categorize counties as shortage areas if there are no mental health specialists of any kind in the county, including psychologists, mental health nurse practitioners, and social workers. We found that this would only identify 16 counties as “shortage areas” which seemed implausibly small.

© 2026 Jorem J et al. *JAMA Network Open*.

Categorizing Mental Health Specialists by Their Uptake of Telemedicine

We defined telemedicine as synchronous two-way video or audio-only services. An outpatient mental health visit was considered a telemedicine visit if the line place of service codes were 02 or 10, the line CPT codes were G2025 (for telemedicine services furnished from rural or federally qualified health centers) or 99441–99443 and 98966–98968 (for audio-only services), or the line modifier codes were GT, GQ, or 95. To ensure our analysis focused on specialists who adopted telemedicine in response to the COVID-19 pandemic, we excluded early adopters who used telemedicine for more than 5% of their outpatient visits before the pandemic.

Visit Characteristics

To characterize the care patterns of the different groups of specialists, we added patient characteristics to each visit from the Master Beneficiary Summary File (MBSF), including patient age at time of visit, female sex, original Medicare entitlement due to disability, and dual Medicaid enrollment. Additionally, we identified visits with a diagnosis code for a substance use disorder (ICD-10 codes F10–19, excluding F17 for smoking cessation) or a diagnosis for serious mental illness, including schizophrenia (ICD-10 codes F20–29) or bipolar I disorder (ICD-10 codes F30, F31.0-31.7) in any diagnosis position on the associated Carrier claim for the visit. The patient's geographic location was based on the first five digits of the beneficiary mailing address zip code on the Carrier claim, reflecting where they received mail at the time of their visit. Note that this differs from the zip code in the MBSF, which is a snapshot updated at the end of each year.

We also added the number of chronic conditions (of 30 total conditions captured by the Chronic Conditions Warehouse algorithms) observed during the calendar year before their visit. These included: acute myocardial infarction, Alzheimer's disease, anemia, asthma, atrial fibrillation and flutter, benign prostatic hyperplasia, cancer (breast), cancer (colorectal), cancer (endometrial), cancer (lung), cancer (prostate), cancer (urologic: kidney, renal pelvis, ureter), cataract, chronic kidney disease, chronic obstructive pulmonary disease (COPD), depression, bipolar, or other depressive mood disorders, diabetes, glaucoma, heart failure and non-ischemic heart disease, hip/pelvic fracture, hyperlipidemia, hypertension, hypothyroidism, ischemic heart disease, non-Alzheimer's dementia, osteoporosis with or without pathological fracture,

© 2026 Jorem J et al. *JAMA Network Open*.

Parkinson’s disease and secondary parkinsonism, pneumonia (all-cause), rheumatoid arthritis/osteoarthritis, stroke/transient ischemic attack (TIA).[2]

[2] Centers for Medicare & Medicaid Services. Chronic Conditions Data Warehouse (CCW): Condition Categories. Centers for Medicare & Medicaid Services. Accessed January 2, 2026. https://www2.ccwdata.org/web/guest/condition-categories

© 2026 Jorem J et al. *JAMA Network Open*.

eTable 1. Linear Regressions of Patients Residing in Mental Health Shortage Areas, Without and With Controlling for Patients Who Moved

	Linear Regression Model					Linear Regression Model Controlling for Patients that Move				
	Coefficient (95% CI)	Bonferroni-Corrected (95% CI)	2018 Mean	Relative Differential Change (95% CI)	Bonferroni-Corrected (95% CI)	Coefficient (95% CI)	Bonferroni-Corrected (95% CI)	2018 Mean	Relative Differential Change (95% CI)	Bonferroni-Corrected (95% CI)
Year indicators										
2019	-0.08 (-0.2, 0.04)		4.01%	-2.05 (-5.06, 0.95)		-0.08 (-0.2, 0.03)		4.01%	-2.09 (-5.04, 0.86)	
2020	0.15 (-0.02, 0.32)		4.01%	3.77 (-0.5, 8.03)		0.17 (0, 0.33)		4.01%	4.11 (-0.06, 8.28)	
2021	0.18 (0, 0.35)		4.01%	4.36 (0.02, 8.7)		0.2 (0.03, 0.37)		4.01%	4.99 (0.81, 9.15)	
2022	0.05 (-0.14, 0.23)		4.01%	1.15 (-3.52, 5.81)		0.05 (-0.13, 0.23)		4.01%	1.24 (-3.22, 5.71)	
2023	0.08 (-0.13, 0.28)		4.01%	1.88 (-3.24, 7.01)		0.08 (-0.12, 0.27)		4.01%	1.92 (-2.87, 6.71)	
Year X telemedicine group indicators										
2019 x Mid-Low	0.1 (-0.06, 0.25)	(-0.13, 0.33)	4.41%	2.18 (-1.29, 5.65)	(-3.01, 7.38)	0.12 (-0.03, 0.26)	(-0.11, 0.34)	4.40%	2.61 (-0.79, 6)	(-2.47, 7.69)
2020 x Mid-Low	-0.07 (-0.28, 0.14)	(-0.39, 0.24)	4.41%	-1.66 (-6.39, 3.08)	(-8.77, 5.45)	-0.05 (-0.25, 0.16)	(-0.35, 0.26)	4.40%	-1.03 (-5.64, 3.59)	(-7.94, 5.88)
2021 x Mid-Low	-0.09 (-0.31, 0.13)	(-0.42, 0.24)	4.41%	-2.09 (-7.07, 2.9)	(-9.54, 5.37)	-0.08 (-0.29, 0.13)	(-0.39, 0.24)	4.40%	-1.75 (-6.55, 3.05)	(-8.94, 5.43)
2022 x Mid-Low	0 (-0.24, 0.24)	(-0.36, 0.36)	4.41%	-0.03 (-5.49, 5.42)	(-8.19, 8.13)	0 (-0.23, 0.23)	(-0.34, 0.35)	4.40%	0.07 (-5.11, 5.27)	(-7.71, 7.85)
2023 x Mid-Low	-0.07 (-0.33, 0.2)	(-0.46, 0.33)	4.41%	-1.48 (-7.44, 4.49)	(-10.42, 7.46)	-0.03 (-0.27, 0.22)	(-0.4, 0.34)	4.40%	-0.65 (-6.23, 4.93)	(-9.01, 7.72)
2019 x Mid-High	0.11 (-0.03, 0.26)	(-0.11, 0.33)	2.78%	4.03 (-1.19, 9.28)	(-3.79, 11.87)	0.1 (-0.04, 0.24)	(-0.11, 0.31)	2.78%	3.71 (-1.39, 8.78)	(-3.92, 11.32)
2020 x Mid-High	-0.1 (-0.31, 0.1)	(-0.41, 0.21)	2.78%	-3.74 (-11.19, 3.74)	(-14.9, 7.45)	-0.12 (-0.32, 0.08)	(-0.43, 0.19)	2.78%	-4.32 (-11.65, 3.01)	(-15.32, 6.66)
2021 x Mid-High	-0.06 (-0.28, 0.16)	(-0.39, 0.27)	2.78%	-2.07 (-10.04, 5.86)	(-13.98, 9.83)	-0.15 (-0.37, 0.07)	(-0.48, 0.18)	2.78%	-5.43 (-13.24, 2.41)	(-17.14, 6.31)
2022 x Mid-High	0.12 (-0.11, 0.36)	(-0.23, 0.48)	2.78%	4.42 (-4.1, 12.99)	(-8.36, 17.23)	0.02 (-0.22, 0.25)	(-0.33, 0.37)	2.78%	0.61 (-7.77, 8.99)	(-11.96, 13.18)
2023 x Mid-High	0.11 (-0.14, 0.36)	(-0.27, 0.49)	2.78%	3.99 (-5.11, 13.09)	(-9.62, 17.63)	-0.01 (-0.25, 0.23)	(-0.37, 0.35)	2.78%	-0.36 (-8.99, 8.27)	(-13.28, 12.56)
2019 x Highest	0.09 (-0.05, 0.22)	(-0.12, 0.29)	1.75%	4.87 (-2.93, 12.69)	(-6.81, 16.56)	0.09 (-0.04, 0.22)	(-0.11, 0.29)	1.74%	5.05 (-2.56, 12.64)	(-6.34, 16.43)
2020 x Highest	-0.01 (-0.21, 0.18)	(-0.3, 0.28)	1.75%	-0.66 (-11.83, 10.51)	(-17.38, 16.06)	-0.04 (-0.23, 0.15)	(-0.33, 0.24)	1.74%	-2.55 (-13.45, 8.33)	(-18.83, 13.73)
2021 x Highest	0 (-0.2, 0.21)	(-0.3, 0.31)	1.75%	0.16 (-11.43, 11.77)	(-17.23, 17.55)	-0.06 (-0.26, 0.13)	(-0.35, 0.23)	1.74%	-3.66 (-14.77, 7.47)	(-20.28, 12.97)
2022 x Highest	0.14 (-0.08, 0.36)	(-0.19, 0.47)	1.75%	7.83 (-4.85, 20.46)	(-11.14, 26.74)	0.08 (-0.13, 0.29)	(-0.23, 0.4)	1.74%	4.78 (-7.3, 16.84)	(-13.3, 22.86)
2023 x Highest	0.12 (-0.13, 0.36)	(-0.25, 0.48)	1.75%	6.8 (-7.14, 20.69)	(-14.08, 27.62)	0.06 (-0.17, 0.29)	(-0.28, 0.4)	1.74%	3.52 (-9.6, 16.67)	(-16.14, 23.17)
Constant	3.71 (3.66, 3.76)					3.72 (3.67, 3.76)				
Observations	28,034,287					28,034,287				
R2	0.347					0.353				
Clinician Fixed-Effects (No.)	17,742					17,742				

Standard errors were clustered at the practice level. Bonferroni-corrected confidence intervals account for 15 comparisons.

© 2026 Jorem J et al. *JAMA Network Open*.

eTable 2. Linear Regressions of Patients Residing in Rural Areas, Without and With Controlling for Patients Who Moved

	Linear Regression Model					Linear Regression Model Controlling for Patients that Move				
	Coefficient (95% CI)	Bonferroni-Corrected (95% CI)	2018 Mean	Relative Differential Change (95% CI)	Bonferroni-Corrected (95% CI)	Coefficient (95% CI)	Bonferroni-Corrected (95% CI)	2018 Mean	Relative Differential Change (95% CI)	Bonferroni-Corrected (95% CI)
Year indicators										
2019	-0.04 (-0.18, 0.11)		12.42%	-0.3 (-1.46, 0.86)		-0.02 (-0.16, 0.11)		12.43%	-0.19 (-1.28, 0.9)	
2020	0.11 (-0.1, 0.32)		12.42%	0.89 (-0.79, 2.56)		0.11 (-0.08, 0.3)		12.43%	0.88 (-0.66, 2.42)	
2021	0.04 (-0.18, 0.27)		12.42%	0.33 (-1.47, 2.13)		0.06 (-0.16, 0.27)		12.43%	0.45 (-1.26, 2.16)	
2022	-0.04 (-0.28, 0.19)		12.42%	-0.36 (-2.24, 1.52)		-0.03 (-0.25, 0.19)		12.43%	-0.22 (-1.98, 1.53)	
2023	-0.16 (-0.42, 0.1)		12.42%	-1.26 (-3.35, 0.81)		-0.16 (-0.39, 0.08)		12.43%	-1.26 (-3.16, 0.63)	
Year X telemedicine group indicators										
2019 x Mid-Low	-0.05 (-0.24, 0.15)	(-0.34, 0.24)	13.98%	-0.34 (-1.73, 1.05)	(-2.42, 1.75)	0.01 (-0.18, 0.19)	(-0.27, 0.28)	13.99%	0.04 (-1.27, 1.34)	(-1.92, 2)
2020 x Mid-Low	-0.14 (-0.41, 0.14)	(-0.55, 0.27)	13.98%	-0.98 (-2.94, 0.98)	(-3.91, 1.96)	-0.07 (-0.32, 0.18)	(-0.45, 0.31)	13.99%	-0.5 (-2.32, 1.31)	(-3.22, 2.21)
2021 x Mid-Low	0.15 (-0.16, 0.45)	(-0.31, 0.61)	13.98%	1.05 (-1.14, 3.25)	(-2.23, 4.34)	0.1 (-0.19, 0.39)	(-0.33, 0.54)	13.99%	0.74 (-1.33, 2.8)	(-2.36, 3.83)
2022 x Mid-Low	0.2 (-0.13, 0.52)	(-0.29, 0.69)	13.98%	1.4 (-0.94, 3.73)	(-2.1, 4.9)	0.12 (-0.19, 0.43)	(-0.35, 0.58)	13.99%	0.83 (-1.39, 3.05)	(-2.49, 4.16)
2023 x Mid-Low	0.27 (-0.09, 0.63)	(-0.26, 0.81)	13.98%	1.95 (-0.62, 4.51)	(-1.89, 5.79)	0.27 (-0.07, 0.6)	(-0.24, 0.77)	13.99%	1.9 (-0.5, 4.3)	(-1.69, 5.49)
2019 x Mid-High	-0.03 (-0.21, 0.15)	(-0.29, 0.24)	10.23%	-0.26 (-2, 1.48)	(-2.87, 2.35)	0 (-0.17, 0.17)	(-0.26, 0.25)	10.24%	-0.02 (-1.68, 1.64)	(-2.5, 2.47)
2020 x Mid-High	-0.05 (-0.31, 0.2)	(-0.43, 0.33)	10.23%	-0.52 (-3.01, 1.97)	(-4.25, 3.22)	-0.01 (-0.25, 0.23)	(-0.36, 0.35)	10.24%	-0.09 (-2.41, 2.24)	(-3.56, 3.39)
2021 x Mid-High	0.12 (-0.16, 0.4)	(-0.3, 0.54)	10.23%	1.18 (-1.57, 3.94)	(-2.95, 5.31)	0.09 (-0.18, 0.36)	(-0.32, 0.49)	10.24%	0.84 (-1.79, 3.48)	(-3.1, 4.78)
2022 x Mid-High	0.23 (-0.07, 0.53)	(-0.21, 0.68)	10.23%	2.26 (-0.65, 5.15)	(-2.09, 6.6)	0.14 (-0.13, 0.42)	(-0.27, 0.56)	10.24%	1.41 (-1.3, 4.11)	(-2.65, 5.46)
2023 x Mid-High	0.3 (-0.03, 0.64)	(-0.2, 0.81)	10.23%	2.97 (-0.31, 6.25)	(-1.94, 7.87)	0.32 (0.01, 0.63)	(-0.15, 0.78)	10.24%	3.13 (0.09, 6.15)	(-1.42, 7.66)
2019 x Highest	-0.02 (-0.2, 0.16)	(-0.29, 0.25)	6.97%	-0.31 (-2.9, 2.27)	(-4.18, 3.55)	-0.04 (-0.21, 0.12)	(-0.3, 0.21)	6.97%	-0.64 (-3.06, 1.76)	(-4.25, 2.96)
2020 x Highest	0.04 (-0.21, 0.3)	(-0.34, 0.42)	6.97%	0.64 (-3, 4.28)	(-4.81, 6.09)	-0.03 (-0.26, 0.21)	(-0.38, 0.33)	6.97%	-0.36 (-3.72, 3)	(-5.38, 4.66)
2021 x Highest	0.45 (0.16, 0.75)	(0.01, 0.9)	6.97%	6.51 (2.28, 10.75)	(0.18, 12.85)	0.2 (-0.07, 0.46)	(-0.2, 0.6)	6.97%	2.84 (-0.97, 6.64)	(-2.87, 8.54)
2022 x Highest	0.68 (0.36, 1)	(0.2, 1.16)	6.97%	9.8 (5.21, 14.35)	(2.92, 16.68)	0.37 (0.08, 0.65)	(-0.06, 0.79)	6.97%	5.24 (1.17, 9.31)	(-0.86, 11.34)
2023 x Highest	0.88 (0.53, 1.22)	(0.36, 1.39)	6.97%	12.55 (7.59, 17.5)	(5.12, 20)	0.55 (0.24, 0.86)	(0.09, 1.01)	6.97%	7.92 (3.5, 12.34)	(1.29, 14.55)
Constant	11.7 (11.7, 11.8)					11.7 (11.7, 11.8)				
Observations	28,034,287					28,034,287				
R2	0.627					0.633				
Clinician Fixed-Effects (No.)	17,742					17,742				

Standard errors were clustered at the practice level. Bonferroni-corrected confidence intervals account for 15 comparisons.

© 2026 Jorem J et al. *JAMA Network Open*.

eTable 3. Linear Regressions of Patients Residing in Different States, Without and With Controlling for Patients Who Moved

	Linear Regression Model					Linear Regression Model Controlling for Patients that Move				
	Coefficient (95% CI)	Bonferroni-Corrected (95% CI)	2018 Mean	Relative Differential Change (95% CI)	Bonferroni-Corrected (95% CI)	Coefficient (95% CI)	Bonferroni-Corrected (95% CI)	2018 Mean	Relative Differential Change (95% CI)	Bonferroni-Corrected (95% CI)
Year indicators										
2019	-0.04 (-0.15, 0.08)		3.46%	-1.03 (-4.45, 2.38)		0 (-0.12, 0.12)		3.50%	0.01 (-3.34, 3.37)	
2020	0.27 (0.1, 0.43)		3.46%	7.69 (2.87, 12.49)		0.25 (0.09, 0.4)		3.50%	7.03 (2.51, 11.54)	
2021	0.56 (0.37, 0.74)		3.46%	16.07 (10.72, 21.45)		0.35 (0.17, 0.52)		3.50%	9.89 (4.94, 14.83)	
2022	0.77 (0.57, 0.96)		3.46%	22.2 (16.53, 27.86)		0.39 (0.21, 0.57)		3.50%	11.09 (5.94, 16.2)	
2023	0.89 (0.68, 1.1)		3.46%	25.61 (19.57, 31.79)		0.41 (0.22, 0.6)		3.50%	11.8 (6.34, 17.26)	
Year X telemedicine group indicators										
2019 x Mid-Low	-0.05 (-0.21, 0.12)	(-0.29, 0.2)	3.38%	-1.38 (-6.27, 3.49)	(-8.69, 5.93)	-0.07 (-0.23, 0.09)	(-0.31, 0.17)	3.44%	-1.92 (-6.6, 2.74)	(-8.91, 5.06)
2020 x Mid-Low	0.08 (-0.15, 0.31)	(-0.27, 0.43)	3.38%	2.37 (-4.47, 9.2)	(-7.86, 12.6)	-0.04 (-0.26, 0.17)	(-0.37, 0.28)	3.44%	-1.27 (-7.59, 5.06)	(-10.74, 8.2)
2021 x Mid-Low	0.28 (0.01, 0.55)	(-0.12, 0.68)	3.38%	8.22 (0.31, 16.15)	(-3.63, 20.09)	0.07 (-0.17, 0.32)	(-0.3, 0.45)	3.44%	2.18 (-5.03, 9.36)	(-8.6, 12.95)
2022 x Mid-Low	0.12 (-0.17, 0.41)	(-0.31, 0.55)	3.38%	3.52 (-4.97, 12.01)	(-9.18, 16.23)	0.01 (-0.26, 0.27)	(-0.39, 0.4)	3.44%	0.16 (-7.53, 7.85)	(-11.36, 11.68)
2023 x Mid-Low	0.04 (-0.26, 0.35)	(-0.41, 0.5)	3.38%	1.27 (-7.72, 10.27)	(-12.19, 14.74)	-0.06 (-0.34, 0.22)	(-0.48, 0.35)	3.44%	-1.83 (-9.94, 6.28)	(-13.98, 10.32)
2019 x Mid-High	0.08 (-0.08, 0.24)	(-0.16, 0.32)	3.42%	2.43 (-2.26, 7.11)	(-4.59, 9.45)	0.04 (-0.11, 0.2)	(-0.19, 0.28)	3.42%	1.3 (-3.22, 5.82)	(-5.47, 8.08)
2020 x Mid-High	0.24 (0.01, 0.47)	(-0.11, 0.58)	3.42%	6.96 (0.21, 13.68)	(-3.15, 17.04)	0.15 (-0.07, 0.36)	(-0.17, 0.47)	3.42%	4.33 (-1.93, 10.61)	(-5.06, 13.73)
2021 x Mid-High	0.38 (0.11, 0.64)	(-0.03, 0.78)	3.42%	10.96 (3.1, 18.83)	(-0.82, 22.76)	0.14 (-0.1, 0.38)	(-0.22, 0.5)	3.42%	4.12 (-2.95, 11.17)	(-6.45, 14.69)
2022 x Mid-High	0.33 (0.04, 0.63)	(-0.11, 0.77)	3.42%	9.68 (1.1, 18.27)	(-3.17, 22.54)	0.09 (-0.17, 0.35)	(-0.3, 0.48)	3.42%	2.73 (-4.88, 10.35)	(-8.69, 14.15)
2023 x Mid-High	0.25 (-0.06, 0.56)	(-0.22, 0.72)	3.42%	7.34 (-1.76, 16.46)	(-6.3, 20.99)	0.08 (-0.21, 0.36)	(-0.34, 0.5)	3.42%	2.23 (-5.99, 10.44)	(-10.07, 14.53)
2019 x Highest	0.07 (-0.11, 0.24)	(-0.19, 0.33)	3.62%	1.91 (-2.9, 6.71)	(-5.28, 9.11)	-0.07 (-0.23, 0.1)	(-0.32, 0.18)	3.62%	-1.81 (-6.41, 2.79)	(-8.72, 5.09)
2020 x Highest	0.28 (0.03, 0.53)	(-0.09, 0.65)	3.62%	7.73 (0.9, 14.56)	(-2.5, 17.96)	-0.05 (-0.28, 0.18)	(-0.39, 0.3)	3.62%	-1.27 (-7.62, 5.08)	(-10.78, 8.24)
2021 x Highest	0.83 (0.53, 1.13)	(0.38, 1.28)	3.62%	22.96 (14.72, 31.22)	(10.61, 35.31)	0.28 (0.01, 0.54)	(-0.12, 0.67)	3.62%	7.62 (0.28, 14.94)	(-3.37, 18.61)
2022 x Highest	0.9 (0.58, 1.23)	(0.41, 1.39)	3.62%	24.92 (15.88, 33.98)	(11.4, 38.43)	0.22 (-0.07, 0.5)	(-0.21, 0.65)	3.62%	5.97 (-1.93, 13.9)	(-5.87, 17.82)
2023 x Highest	0.95 (0.6, 1.3)	(0.43, 1.48)	3.62%	26.33 (16.6, 35.91)	(11.76, 40.88)	0.29 (-0.02, 0.6)	(-0.18, 0.76)	3.62%	8.01 (-0.59, 16.63)	(-4.87, 20.92)
Constant	3.5 (3.43, 3.57)					3.54 (3.48, 3.6)				
Observations	28,034,287					28,034,287				
R2	0.154					0.163				
Clinician Fixed-Effects (No.)	17,742					17,742				

Standard errors were clustered at the practice level. Bonferroni-corrected confidence intervals account for 15 comparisons.

© 2026 Jorem J et al. *JAMA Network Open*.

eTable 4. Linear Regressions of Patients Residing 20 Miles or More Away, Without and With Controlling for Patients Who Moved

	Linear Regression Model					Linear Regression Model<br>Controlling for Patients that Move				
	Coefficient (95% CI)	Bonferroni-Corrected (95% CI)	2018 Mean	Relative Differential Change (95% CI)	Bonferroni-Corrected (95% CI)	Coefficient (95% CI)	Bonferroni-Corrected (95% CI)	2018 Mean	Relative Differential Change (95% CI)	Bonferroni-Corrected (95% CI)
Year indicators										
2019	0 (-0.26, 0.26)		16.44%	-0.02 (-1.58, 1.55)		-0.07 (-0.31, 0.16)		16.47%	-0.45 (-1.9, 1)	
2020	0.76 (0.42, 1.11)		16.44%	4.64 (2.53, 6.75)		0.58 (0.25, 0.91)		16.47%	3.5 (1.51, 5.49)	
2021	1.65 (1.26, 2.04)		16.44%	10.04 (7.66, 12.41)		1.02 (0.65, 1.39)		16.47%	6.19 (3.95, 8.44)	
2022	2.44 (1.99, 2.89)		16.44%	14.84 (12.1, 17.58)		1.45 (1.02, 1.88)		16.47%	8.8 (6.19, 11.41)	
2023	2.7 (2.2, 3.2)		16.44%	16.42 (13.38, 19.46)		1.52 (1.04, 2)		16.47%	9.23 (6.31, 12.14)	
Year X telemedicine group indicators										
2019 x Mid-Low	0.1 (-0.24, 0.44)	(-0.41, 0.6)	16.45%	0.6 (-1.45, 2.66)	(-2.47, 3.68)	0.07 (-0.24, 0.39)	(-0.4, 0.54)	16.53%	0.44 (-1.47, 2.34)	(-2.42, 3.29)
2020 x Mid-Low	0.51 (0.04, 0.97)	(-0.2, 1.21)	16.45%	3.07 (0.22, 5.92)	(-1.2, 7.34)	0.23 (-0.2, 0.66)	(-0.42, 0.88)	16.53%	1.4 (-1.21, 4.02)	(-2.51, 5.32)
2021 x Mid-Low	0.88 (0.35, 1.4)	(0.09, 1.67)	16.45%	5.33 (2.13, 8.51)	(0.53, 10.13)	0.62 (0.13, 1.11)	(-0.11, 1.35)	16.53%	3.74 (0.78, 6.72)	(-0.69, 8.17)
2022 x Mid-Low	0.32 (-0.27, 0.91)	(-0.56, 1.2)	16.45%	1.94 (-1.62, 5.5)	(-3.4, 7.27)	0.25 (-0.3, 0.81)	(-0.57, 1.08)	16.53%	1.54 (-1.81, 4.88)	(-3.47, 6.54)
2023 x Mid-Low	0.31 (-0.34, 0.96)	(-0.67, 1.28)	16.45%	1.87 (-2.09, 5.82)	(-4.05, 7.79)	0.34 (-0.28, 0.96)	(-0.59, 1.26)	16.53%	2.05 (-1.69, 5.79)	(-3.55, 7.65)
2019 x Mid-High	0.08 (-0.25, 0.41)	(-0.41, 0.57)	15.75%	0.51 (-1.56, 2.58)	(-2.59, 3.62)	0.13 (-0.18, 0.43)	(-0.33, 0.59)	15.75%	0.8 (-1.15, 2.76)	(-2.12, 3.73)
2020 x Mid-High	0.48 (0.03, 0.93)	(-0.19, 1.15)	15.75%	3.06 (0.21, 5.91)	(-1.2, 7.33)	0.34 (-0.09, 0.77)	(-0.3, 0.99)	15.75%	2.18 (-0.55, 4.91)	(-1.91, 6.27)
2021 x Mid-High	1.13 (0.6, 1.65)	(0.33, 1.92)	15.75%	7.17 (3.8, 10.48)	(2.13, 12.18)	0.71 (0.21, 1.21)	(-0.04, 1.46)	15.75%	4.5 (1.33, 7.68)	(-0.25, 9.26)
2022 x Mid-High	1.1 (0.49, 1.71)	(0.19, 2.02)	15.75%	6.98 (3.11, 10.86)	(1.18, 12.8)	0.67 (0.09, 1.25)	(-0.2, 1.54)	15.75%	4.24 (0.54, 7.94)	(-1.3, 9.79)
2023 x Mid-High	1.37 (0.69, 2.05)	(0.36, 2.39)	15.75%	8.7 (4.41, 13.02)	(2.27, 15.16)	1.05 (0.41, 1.7)	(0.08, 2.02)	15.75%	6.67 (2.58, 10.79)	(0.53, 12.85)
2019 x Highest	0.19 (-0.15, 0.52)	(-0.32, 0.69)	13.22%	1.42 (-1.12, 3.96)	(-2.39, 5.23)	0.03 (-0.28, 0.34)	(-0.44, 0.5)	13.17%	0.24 (-2.13, 2.6)	(-3.3, 3.78)
2020 x Highest	0.83 (0.37, 1.3)	(0.13, 1.53)	13.22%	6.29 (2.76, 9.83)	(1, 11.57)	0.19 (-0.24, 0.62)	(-0.46, 0.83)	13.17%	1.43 (-1.85, 4.7)	(-3.48, 6.33)
2021 x Highest	1.94 (1.4, 2.49)	(1.13, 2.76)	13.22%	14.67 (10.59, 18.84)	(8.55, 20.87)	0.95 (0.45, 1.45)	(0.21, 1.7)	13.17%	7.24 (3.45, 11.01)	(1.56, 12.91)
2022 x Highest	2.24 (1.62, 2.86)	(1.31, 3.17)	13.22%	16.94 (12.25, 21.63)	(9.87, 23.99)	1.07 (0.5, 1.65)	(0.21, 1.93)	13.17%	8.12 (3.78, 12.53)	(1.61, 14.67)
2023 x Highest	2.62 (1.94, 3.3)	(1.6, 3.64)	13.22%	19.82 (14.67, 24.96)	(12.1, 27.56)	1.45 (0.82, 2.09)	(0.5, 2.4)	13.17%	11.01 (6.19, 15.87)	(3.79, 18.23)
Constant	16.1 (15.9, 16.2)					16.1 (16, 16.2)				
Observations	28,034,287					28,034,287				
R2	0.276					0.29				
Clinician Fixed-Effects (No.)	17,742					17,742				

Standard errors were clustered at the practice level. Bonferroni-corrected confidence intervals account for 15 comparisons.

© 2026 Jorem J et al. *JAMA Network Open*.

eFigure 1. Flowchart of Mental Health Specialist Cohort Exclusions, 2018-2023

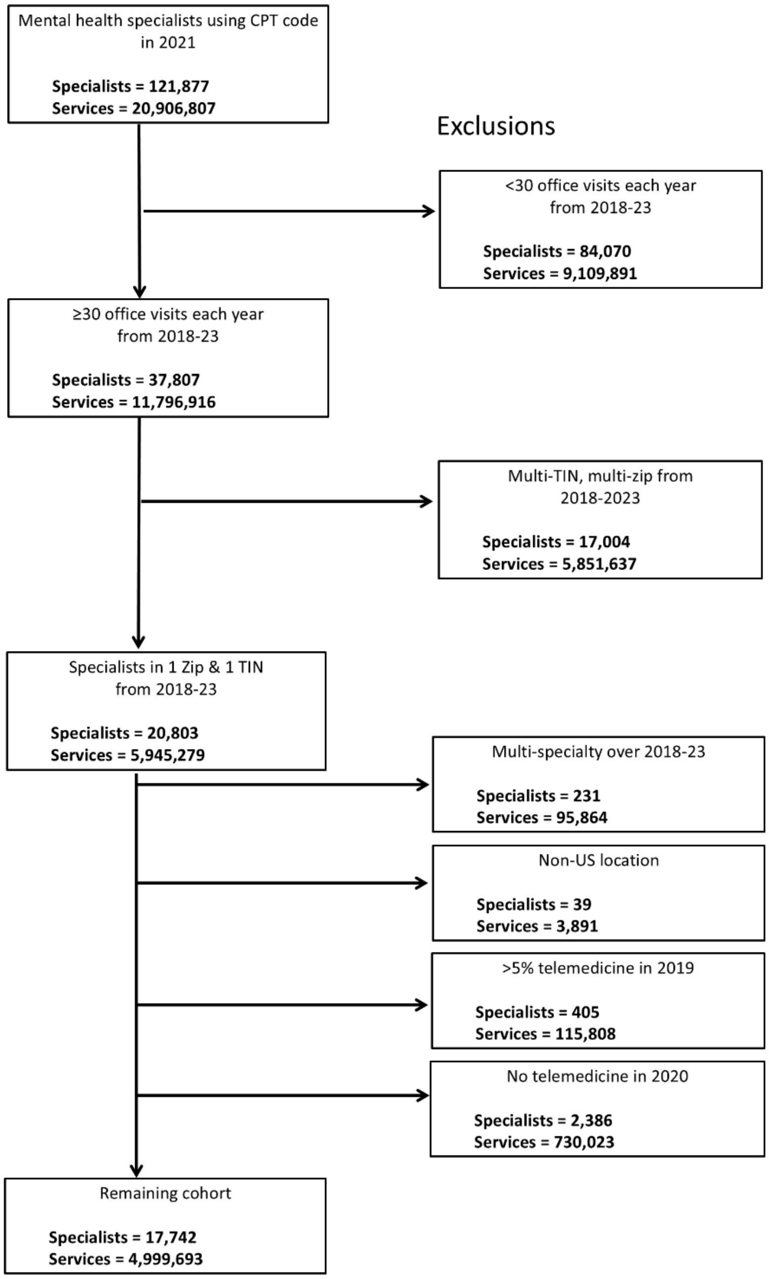

The flowchart shows the number of specialists and MH services in 2021 that were excluded after each sample creation step we took. Our first exclusion, requiring at least 30 outpatient visits every year, was the costliest in terms of sample, removing roughly 70% of specialists. Yet, the remaining 30% of specialists delivered over half (56%) of the mental health services to traditional Medicare in 2021. In **eFigure 7** below, we show changes in our study outcomes among specialists from this 30%, including the multi-TIN and multi-zip specialists.

© 2026 Jorem J et al. *JAMA Network Open*.

eFigure 2. Distribution of Telemedicine Use by Mental Health Specialists in 2021

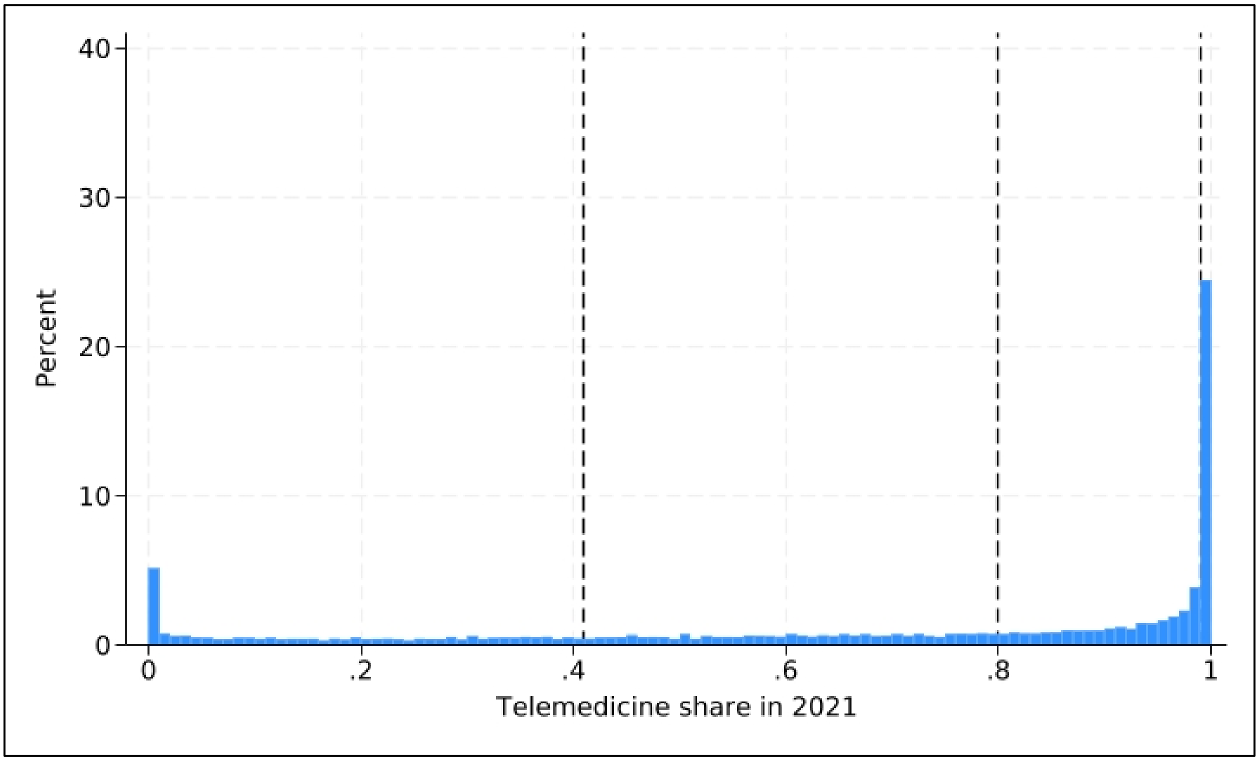

Distribution of eligible mental health specialists across four quartiles, indicated by dotted lines, based on the percentage of their outpatient mental health visits delivered via telemedicine in 2021: lowest use (0–40%), low-mid use (41–79%), mid-high use (80–98%), and highest use (99–100%). Given the skewed, bimodal distribution of the percentage of visits delivered via telemedicine in 2021, we categorized practices into quartiles to ensure adequate sample sizes within each group. As a sensitivity analysis (**eFigure 6**), we also used alternative categories based on predefined cutoffs: 0–15% (minimal use), 16–50% (moderate use), 51–89% (majority use), and 90–100% (high use).

© 2026 Jorem J et al. *JAMA Network Open*.

eFigure 3. Telemedicine Share of Monthly Visits by 2021 Telemedicine Quartile Group Assignment, 2018-2023

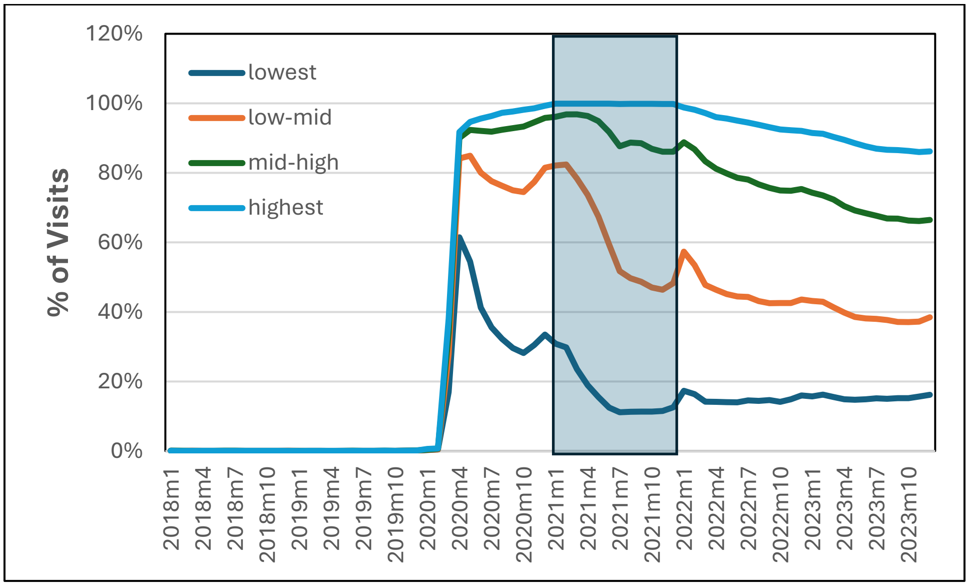

The plot shows the trend in telemedicine share for each quartile group assigned in 2021. The shaded region highlights the time period used for assignment.

© 2026 Jorem J et al. *JAMA Network Open*.

eFigure 4. Monthly Unadjusted Trends in Outcomes From 2018 to 2023 by Mental Health Specialists' Level of Telemedicine use in 2021, Shown as Percentage Point Differences Relative to January 2018 Levels

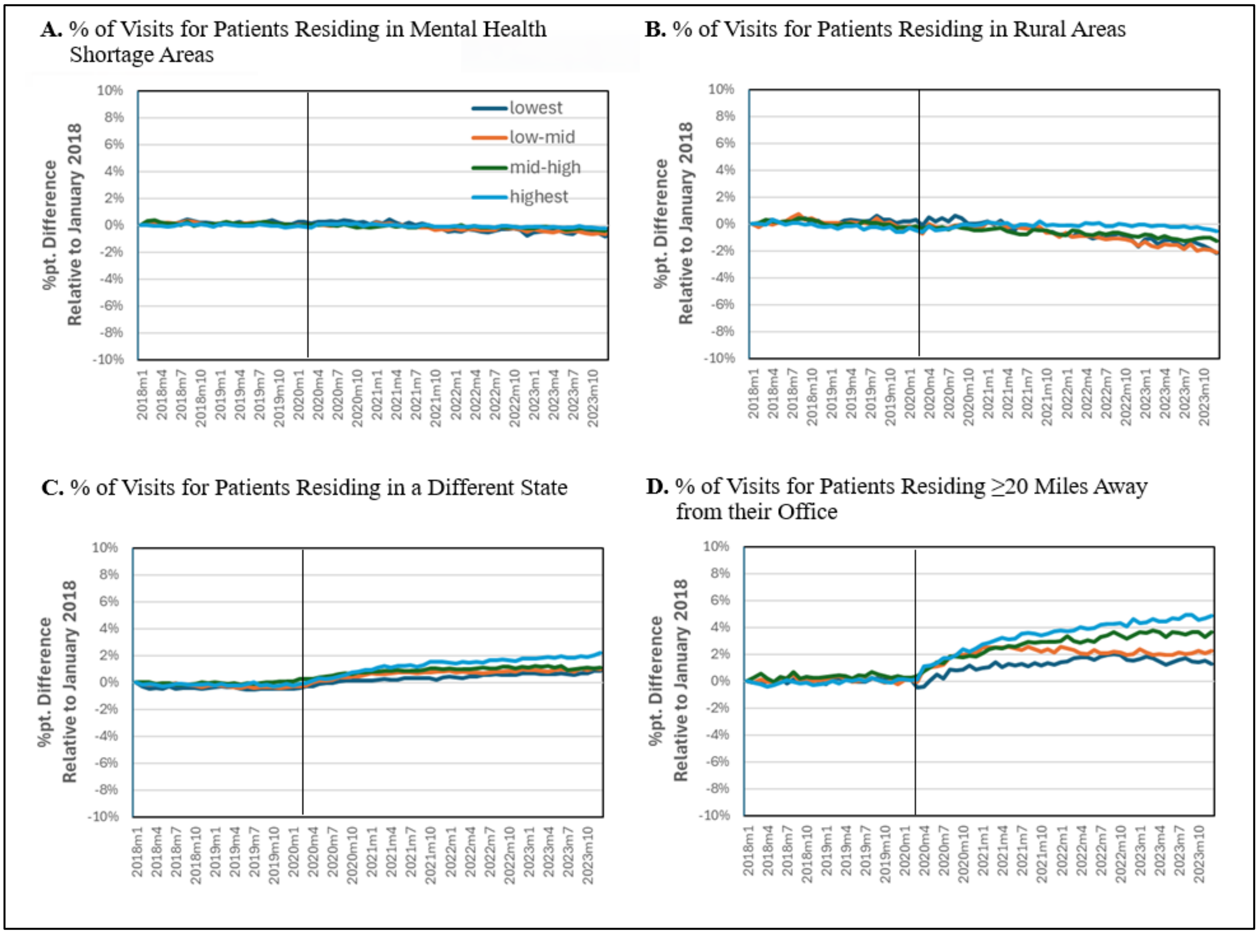

Each panel describes trends in monthly differences between each telemedicine group's unadjusted outcome average compared to the first month of our study period, January 2018. We added these difference plots to better illustrate the magnitude of the changes in the geographic reach of clinicians compared to their reach in the pre-period, as well as to make the changes more comparable across outcomes. The vertical line represents the start of the COVID-19 pandemic in March 2020.

© 2026 Jorem J et al. *JAMA Network Open*.

eFigure 5. Kernel Density Plots of the Distribution of Log-Transformed Miles Between Patients and Specialists Among the Lowest and Highest Telemedicine Users in 2018 and 2023

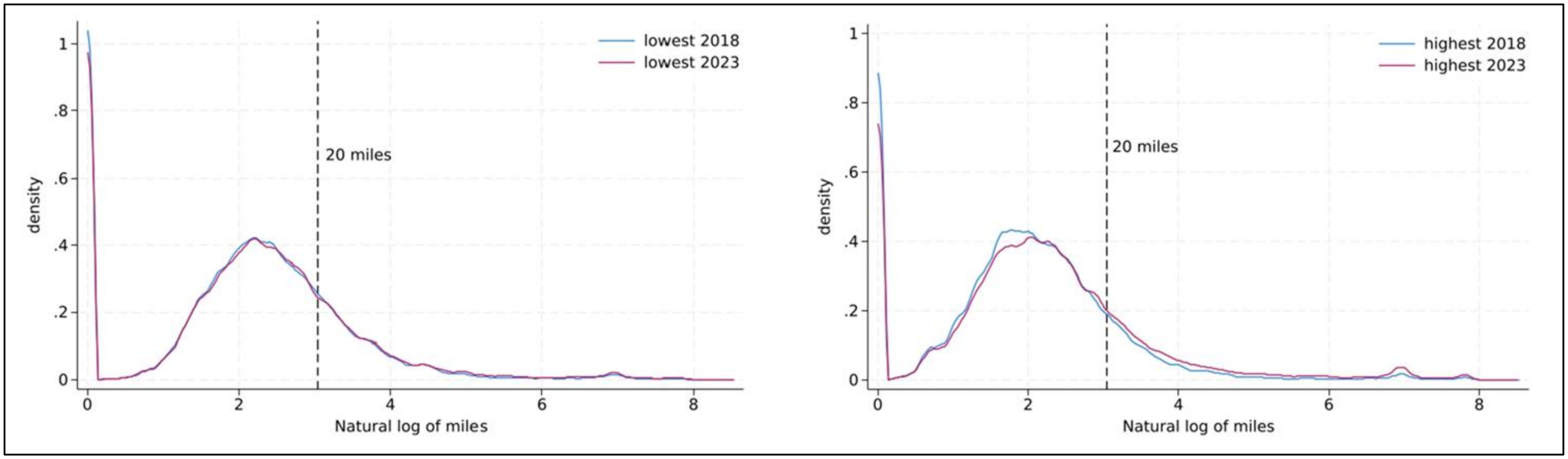

<u>Sample Sizes</u>

Lowest telemedicine
2018 = 1,265,679
2023 = 1,127,689

Highest telemedicine
2018 = 1,012,312
2023 = 978,804

Kernel density estimates visualize the distribution of log transformed patient-specialist distances (in miles) among the lowest and highest telemedicine users in 2018 and 2023. In this secondary analysis, we did not apply a 20-mile threshold (indicated by the dotted line), but instead examined the distribution of distances across all visits. Among specialists with the lowest telemedicine use (left panel), the distribution remains largely unchanged between 2018 and 2023. Specialists with the highest telemedicine adoption (right panel) show a slight shift toward greater distances by 2023.

© 2026 Jorem J et al. *JAMA Network Open*.

eFigure 6. Differential Changes in Outcomes Between Highest and Lowest Telemedicine Groups Using Quartiles or Predefined Cutoffs

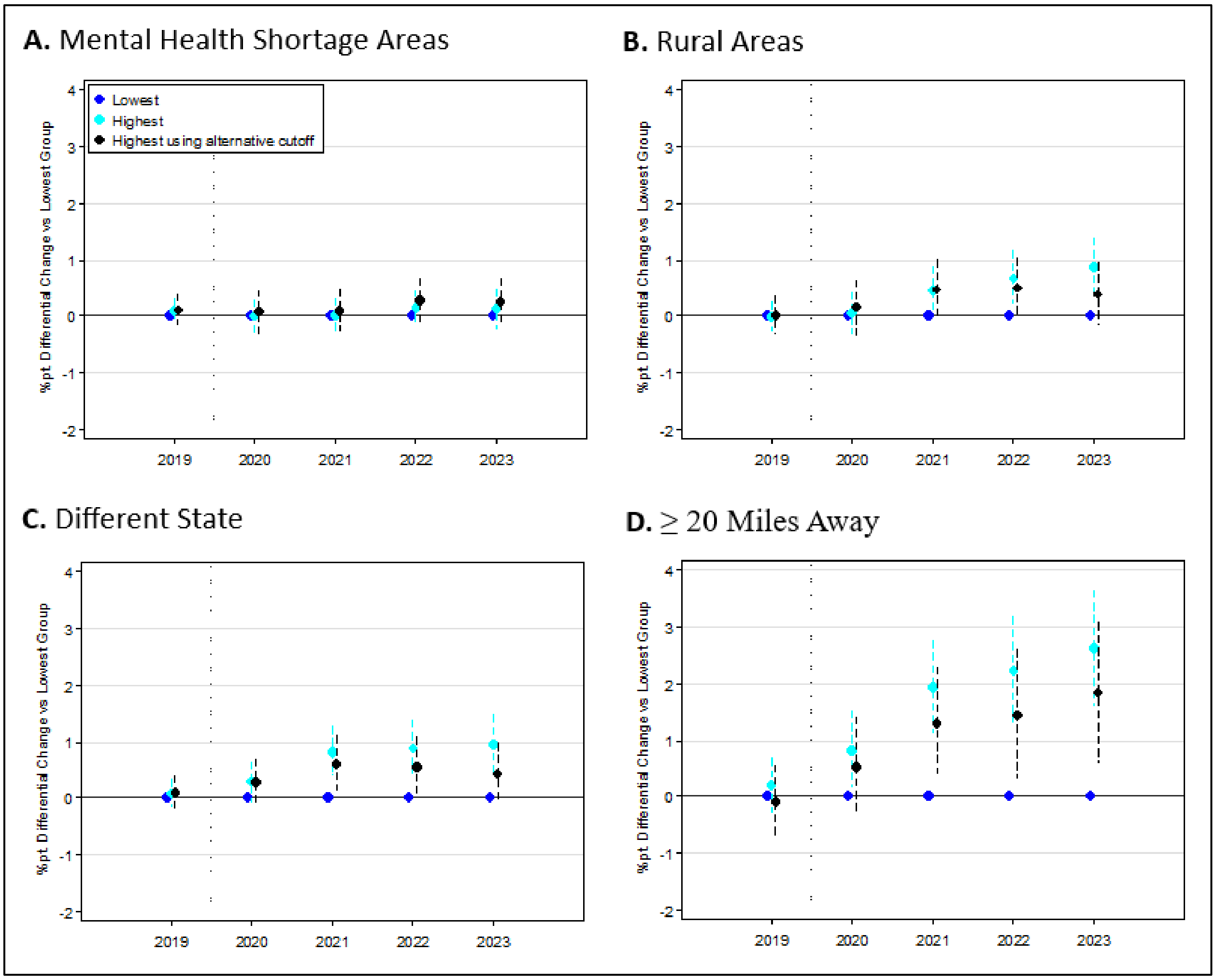

In our main analysis we divided specialists into evenly sized quartiles based on their telemedicine use in 2021. eFigure 2 shows the distribution of telemedicine use in 2021 and the cutoffs we used. We considered an alternative method based on predefined cutoffs: 0–15% (minimal use), 16–50% (moderate use), 51–89% (majority use), and 90–100% (high use).

eFigure 4 above shows our main analysis estimates for the highest group (in light blue) alongside estimates for the predefined cutoff of 90-100% as the highest group (in black). To be more specific, in our main analysis, the highest vs. lowest estimates compared specialists with 99-100% telemedicine to specialists with 0-40% telemedicine, respectively. In this sensitivity, the highest vs. lowest estimates compared specialists with 90-100% telemedicine to specialists with 0-15% telemedicine.

We found that estimates were slightly smaller using these predefined cutoffs to categorize clinicians, but substantively the same as the estimates using evenly sized quartiles.

© 2026 Jorem J et al. *JAMA Network Open*.

eFigure 7. Differential Changes in Outcomes Between the Highest and Lowest Telemedicine Use Specialists, Using a Larger Sample of Specialists

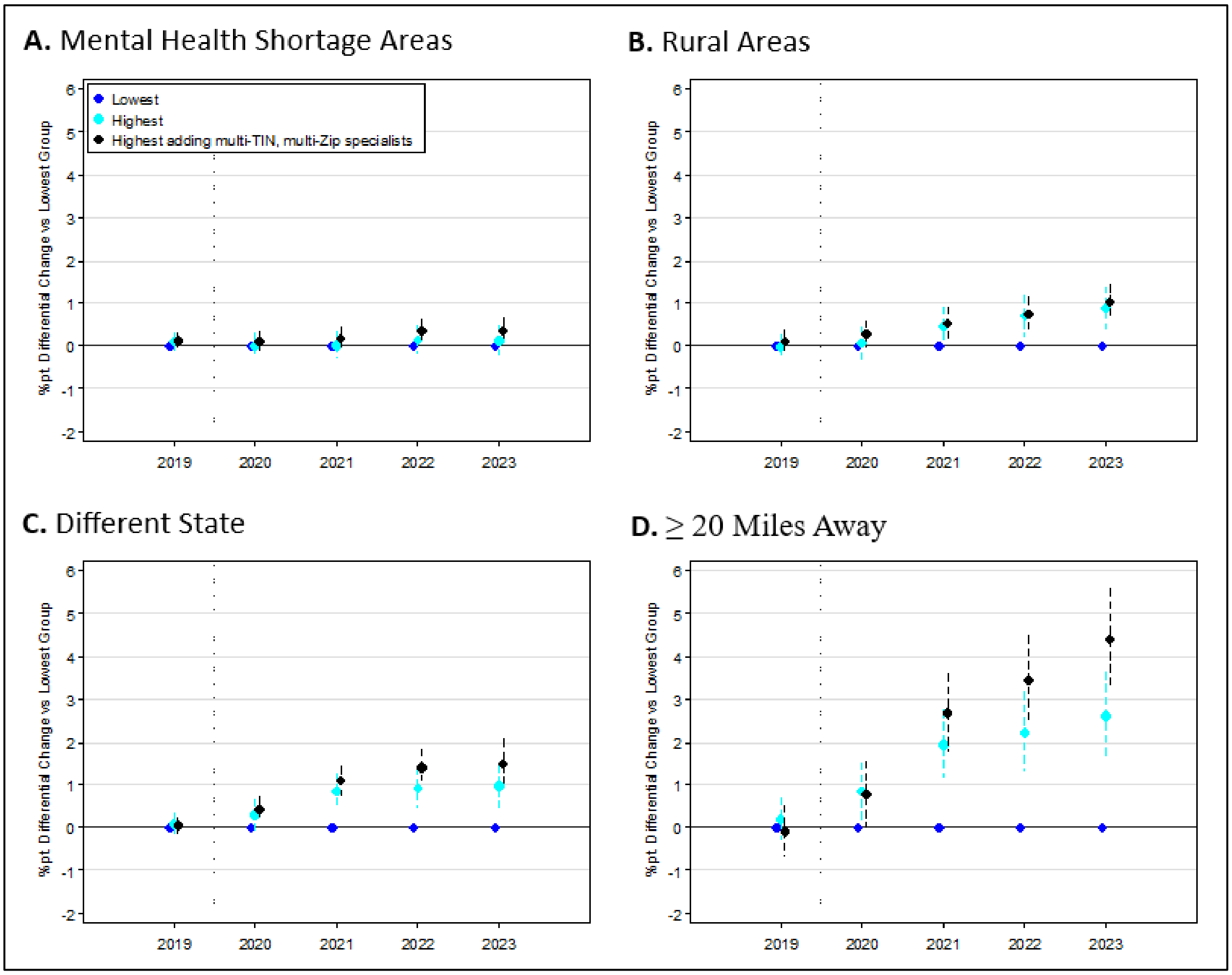

In our main analysis, we focused on specialists that worked in 1 practice and from 1 zip code over our study period. This helped us better understand the changes in georeach outcomes we observed. Yet, excluding multi-TIN or multi-Zip specialists removed roughly half of the specialists who were otherwise qualified to be included in our study (see **eFigure 1** above for the sample flowchart).

eFigure 5 shows what happens to our highest vs lowest telemedicine use estimates when we skip this exclusion and allow multi-TIN or multi-Zip specialists in our study sample. The estimates from our main analysis are shown in light blue and the estimates using both the single and multi TIN/Zip specialists are shown in black. The figure shows that both samples yield substantively similar results, though the changes for different state and 20+ miles are larger when including specialists who move over the study period. These higher estimates were attributed to the clinicians moving and maintaining continuity with their existing panels.

© 2026 Jorem J et al. *JAMA Network Open*.

eFigure 8. Differential Changes in Outcomes Between the Highest and Lowest Telemedicine Use Specialists, Using an Analytic Sample of 1 Observation Per Patient-Specialist Per Year

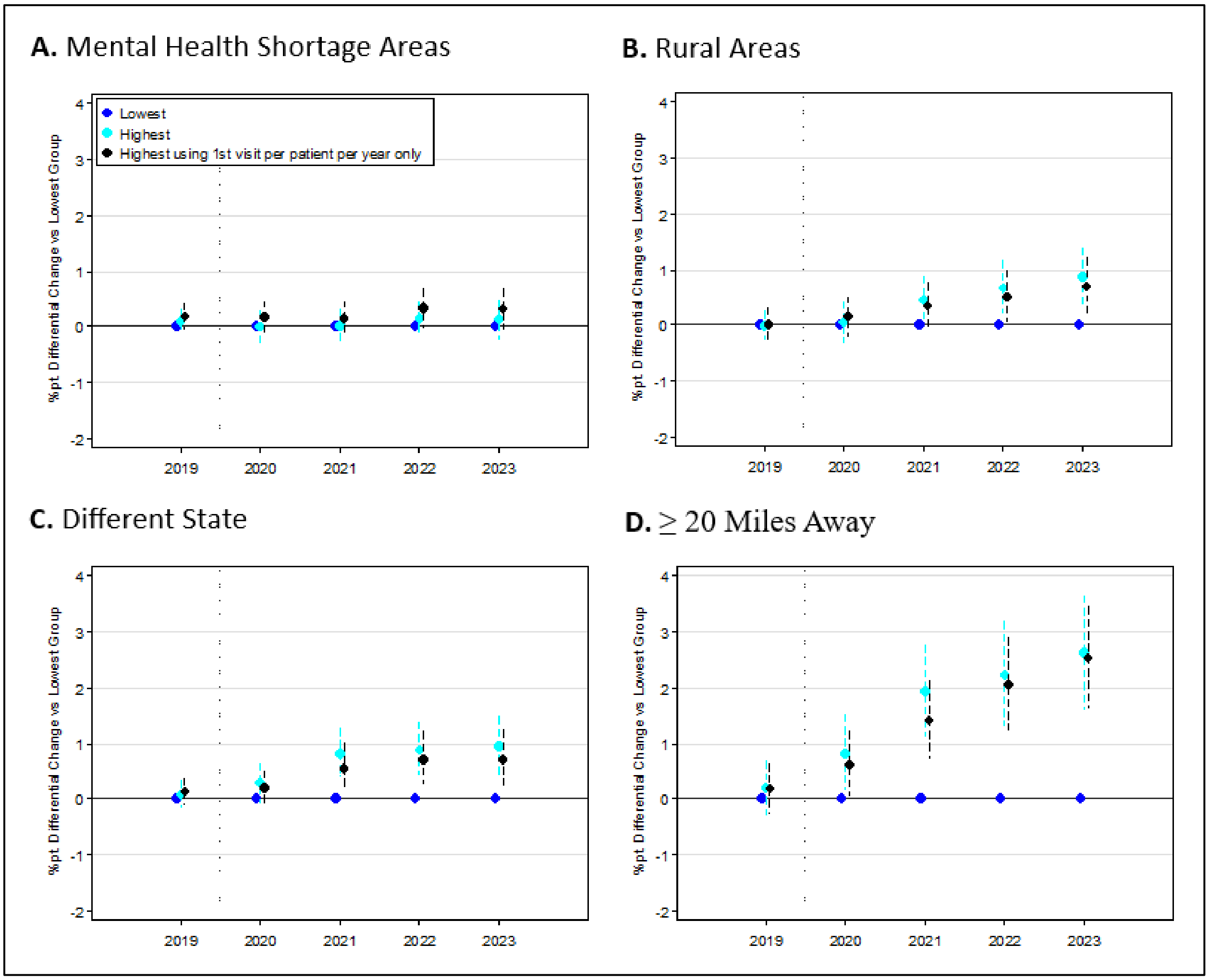

Our main analysis was at the specialist-visit level. In this sensitivity, we kept the first visit per patient-specialist in each year and estimated our models using this data. This is the same data used for **Figure 4**, but here we show that our geographic area outcome results are similar when we limit the influence of each patient to just 1 observation per specialist-year.

© 2026 Jorem J et al. *JAMA Network Open*.

eFigure 9. Differential Changes in Patients Living 10, 20, or 30 Miles or More From Their Specialist Between Highest and Lowest Telemedicine Groups

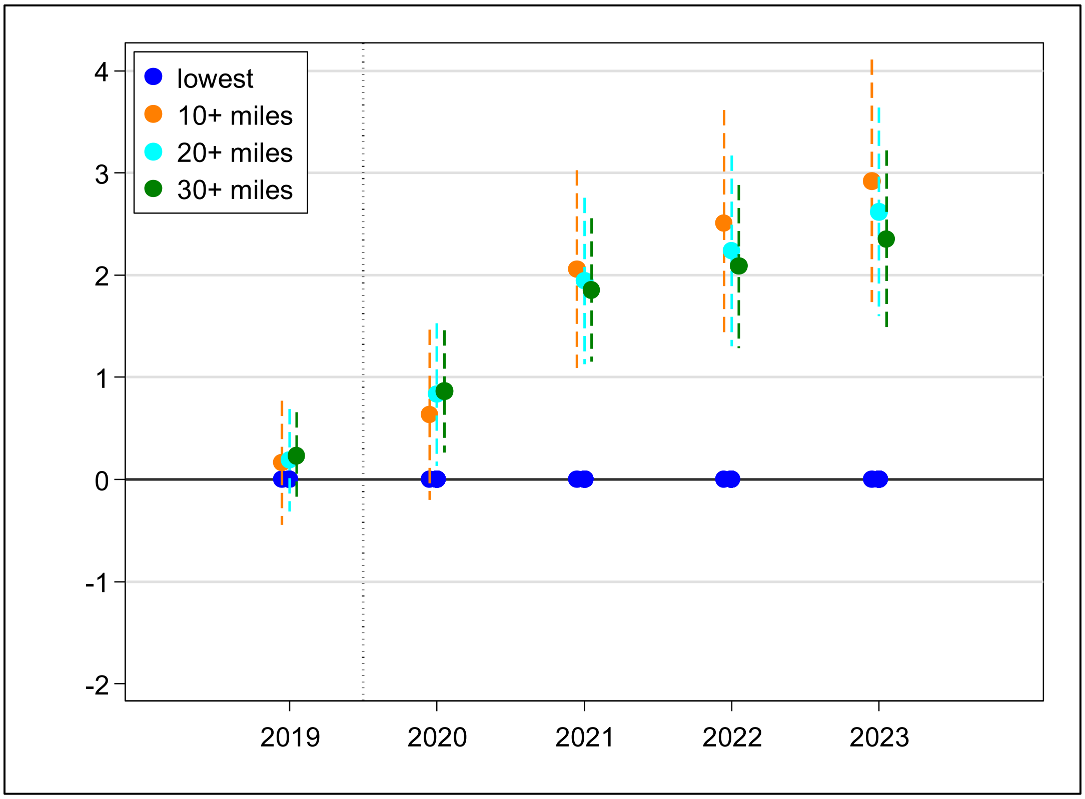

Instead of using 20 miles as the threshold for evaluating far distances between patients and specialists, we estimated differential changes using alternative distances to define this outcome. eFigure 6 shows the estimates from these sensitivities together: using 10+ miles as the threshold, 20+ miles (what we used in our main analysis), and 30+ miles as the threshold. We only show the estimates from the highest vs lowest groups.

While not exactly the same, we found that our results are consistent across these different definitions of distance between patients and specialists.

© 2026 Jorem J et al. *JAMA Network Open*.

eReferences

1. Richard JV, Huskamp HA, Barnett ML, Busch AB, Mehrotra A. A methodology for identifying behavioral health advanced practice registered nurses in administrative claims. *Health Services Research*. 2022:973-978. doi:10.1111/1475-6773.13974
2. Busch AB, Huskamp HA, Raja P, Rose S, Mehrotra A. Disruptions in Care for Medicare Beneficiaries With Severe Mental Illness During the COVID-19 Pandemic. *JAMA Netw Open*. Jan 4 2022;5(1):e2145677. doi:10.1001/jamanetworkopen.2021.45677
3. Centers for Medicare & Medicaid Services. Chronic Conditions Data Warehouse (CCW): Condition Categories. Centers for Medicare & Medicaid Services. Accessed January 2, 2026. https://www2.ccwdata.org/web/guest/condition-categories

© 2026 Jorem J et al. *JAMA Network Open*.
