# Supplementary material for: Mental Health Specialist Telemedicine Uptake and Patient Location
Source: JAMA Netw Open. 2026 Mar 5;9(3):e260823. doi: 10.1001/jamanetworkopen.2026.0823 (PMC12964166; doi:10.1001/jamanetworkopen.2026.0823)
Supplement: Supplement 2. — Data Sharing Statement [file jamanetwopen-e260823-s002.pdf]

Data Sharing Statement

Data
Data available: No

Additional Information
Explanation for why data not available: Data were obtained via a data use agreement with Medicare which precludes us from sharing it with other researchers.
